# Accelerating Mountain Forest Dynamics in the Alps

**DOI:** 10.1007/s10021-021-00674-0

**Published:** 2021-07-28

**Authors:** Dominik Thom, Rupert Seidl

**Affiliations:** 1 Ecosystem Dynamics and Forest Management Group, School of Life Sciences, Technical University of Munich, Hans-Carl-von-Carlowitz-Platz 2, 85354 Freising, Germany; 2 Department of Forest- and Soil Sciences, Institute of Silviculture, University of Natural Resources and Life Sciences (BOKU) Vienna, Peter-Jordan-Straße 82, 1190 Vienna, Austria; 3 Gund Institute for Environment, University of Vermont, 617 Main Street, Burlington, Vermont 05405 USA; 4 Berchtesgaden National Park, Doktorberg 6, 83471 Berchtesgaden, Germany

**Keywords:** climate change, composition, disturbance, forest development, forest dynamics, forest structure, Berchtesgaden National Park, site conditions

## Abstract

Climate change alters forest development pathways, with consequences for ecosystem services and biodiversity. As the rate of warming increases, ecosystem change is expected to accelerate. However, ecosystem dynamics can have many causes unrelated to climate (for example, disturbance and stand development legacies). The compound effects of multiple drivers remain largely unclear. Here, we assessed forest dynamics over 28 years at Berchtesgaden National Park (BGNP), Germany, quantifying the spatiotemporal patterns and unraveling the drivers of forest change. We analyzed high-density forest inventory data, consisting of three consecutive censuses of 3759 permanent sample plots (132,866 tree records in total). We used semi-variograms to analyze spatial patterns of change, and boosted regression trees to quantify the effect of 30 covariates on changes in nine indicators of forest structure and composition. Over the 28 years investigated, the forests of BGNP were becoming denser, structurally more complex, and more species rich. Changes in forest structure were more pronounced and spatially correlated on the landscape than changes in tree species composition. Change rates of all indicators increased over time, signifying an acceleration of forest dynamics since the 1980s. Legacies and climate were the most important drivers of change, but had diverging impacts. Although forest change accelerated with increasing temperature, high legacy levels typical for late development stages dampened it. We here provide evidence for accelerating forest dynamics in mountain forests of the Alps, with potentially far-reaching consequences for biodiversity and ecosystem processes. We highlight that unmanaged forest development toward old-growth conditions could counteract climate-mediated acceleration of forest change.

**Supplementary Information:**

The online version contains supplementary material available at 10.1007/s10021-021-00674-0.

## Highlights

- Forests have become denser, structurally more complex, and more species-rich
- Increasing temperature has accelerated forest change
- Late development stages dampen climate effects on forest change

## Introduction

Climate change and increases in disturbance activity are already altering forest ecosystem dynamics (Anderegg and others 2013; Dhar and others 2016). As a consequence of these ongoing changes, studies predict a decrease in the supply of ecosystem services essential for human well-being (Schröter and others 2005; Seidl and others 2019). Understanding the development trajectories and climate sensitivities of forest ecosystems is thus of paramount importance. Studying the impact of recent changes in climate provides an opportunity to better understand forest ecosystem dynamics. It furthermore allows the identification of the main drivers of recent forest change. Multi-decadal records of ecosystem change are increasingly available for such analyses (see, for example, Boucher and others 2021) and can provide detailed insights into the intricacies of forest dynamics, addressing questions such as: what is the role of climate in recent forest change, and how are legacies, disturbance, demography, and topography modulating climate-induced changes in forest structure and composition.

Site conditions, including topography and soils, determine local climate and resource availability and thus regulate ecosystem dynamics (Seddon and others 2016). Hence, site conditions act as environmental filters for forest composition and structure (Chapman and McEwan 2018) and can either amplify or dampen the effects of climate change. Topographic complexity may, for instance, buffer climate change impacts on forest ecosystems and delay tipping points toward alternative stable states (Albrich and others 2020), with tipping points being nonlinear and potentially irreversible ecosystem alterations (Reyer and others 2015). However, topographic exposure can also elevate the propensity for natural disturbances (Mitchell 2013), and shallow soils with low water-holding capacity can further amplify the effect of hotter drought (Paz-Kagan and others 2017). As a consequence, the impacts of climate change on ecosystem dynamics vary strongly in space and time and could entail tipping points, especially if ecosystems are not well buffered and/ or multiple drivers change simultaneously (Turner and others 2020). For instance, climate change has accelerated forest productivity in Central European forests since 1870 (Pretzsch and others 2014), but may dampen growth rates in the future as a result of increasing temperature and water stress (Hoffmann and others 2018).

Natural disturbance regimes are expected to intensify globally as a consequence of climate change (Seidl and others 2017). Disturbances alter successional and structural development pathways (Meigs and others 2017). However, disturbance impacts on forest ecosystems vary strongly depending on disturbance characteristics. Disturbances with moderate frequency and severity may catalyze the adaptation of ecosystems to novel environmental conditions, and foster ecosystem heterogeneity and biodiversity (Thom and others 2017a, b; Sommerfeld and others 2020). In contrast, large-scale high-severity disturbances may lead to biotic homogenization (Thom and others 2017a; Senf and others 2020), further eroding the ability of ecosystems to respond to global change (Mori and others 2018). Recent “mega disturbances”, including, for instance, fires in Australia, California, and Siberia, as well as bark beetle outbreaks in western North America and Central Europe, are facilitated by climate change (Sambaraju and others 2019; Ward and others 2020) and are expected to become more prominent in the future (Millar and Stephenson 2015). Also human land use has been an important driver of ecosystem change around the globe (McDowell and others 2020). Most European forests have been managed for centuries, with long-lasting effects on their past and future development (Bürgi and others 2017). Such legacies of historic land use strongly determine current ecosystem dynamics (Stritih and others 2021), and may even be a more influential driver of forest change throughout the twenty-first century than changing climate and disturbance regimes (Thom and others 2018).

Disturbances and land-use strongly alter the demography of forest ecosystems (Kulakowski and others 2017), and their legacies lay the foundation for future ecosystem development. For instance, older forests are oftentimes associated with higher ecosystem complexity, resulting in higher response diversity that can buffer them against environmental change and disturbance (Donato and others 2012; Meigs and others 2017; Urbano and Keeton 2017). Furthermore, young forests have been shown to respond more strongly to altered environmental conditions compared to old forests, and are, for instance, better able to utilize elevated atmospheric CO_2_ concentrations (Norby and others 2016). As a consequence, forest development pathways under novel environmental conditions will likely depend on forest age (Anderson-Teixeira and others 2013).

To better understand the intricacies of ongoing forest change, we here investigated forest dynamics at Berchtesgaden National Park (BGNP) in southeastern Germany. BGNP is strictly protected since 1978 and represents the high topographic complexity and environmental heterogeneity that is typical for mountain forests of the Alps (Thom and others 2017a; Senf and Seidl 2018). Before being designated as a national park, the landscape experienced centuries of intensive land-use, similar to many other landscapes in the area (see for example, Thom and others 2018). After an intensification in salt mining in the sixteenth century the local wood demand increased dramatically, leading to large clear-cuts and an exploitation of the area (Zierl 2009). After political changes in the early nineteenth-century reforestation projects were initiated, mainly promoting naturally occurring conifer species including Norway spruce (*Picea abies* [K.]) and European larch (*Larix decidua* [Mill.]). Regeneration of other native tree species often failed due to high grazing pressure. The initial protection of parts of the landscape was enacted already more than 110 years ago, eventually leading to the foundation of the national park in 1978. In the past decades, management focused on restoration activities in the management zone of the national park (25% of the area). The impact of natural disturbances was only moderate in the past decades (Senf and Seidl 2018).

Here, we base our investigation on a high-density network of permanent forest inventory plots (with more than 43 plots per 100 ha of forest area) that were censused three times over a period of 28 years. Our objectives were (1) to assess spatiotemporal changes in forest structure and composition and (2) to unravel the underlying drivers of forest change. We hypothesized an acceleration of forest dynamics due to climate change, as the Alps are particularly exposed to ongoing warming (Engler and others 2011). In particular, we expected forest structure to change faster than composition, as structure can respond immediately to climatic extremes (for example, through elevated tree mortality), while tree species compositions reassemble only over a period of decades to centuries in the mountain forests of the Alps (Thom and others 2017b; Albrich and others 2020). Further, we hypothesized disturbances to catalyze changes in forest ecosystems, but topographic complexity and high levels of past legacies (for example, complex structures resulting from past disturbance and stand development) to buffer ecosystem alterations.

## Materials and Methods

### Study Area

**Figure 1:**
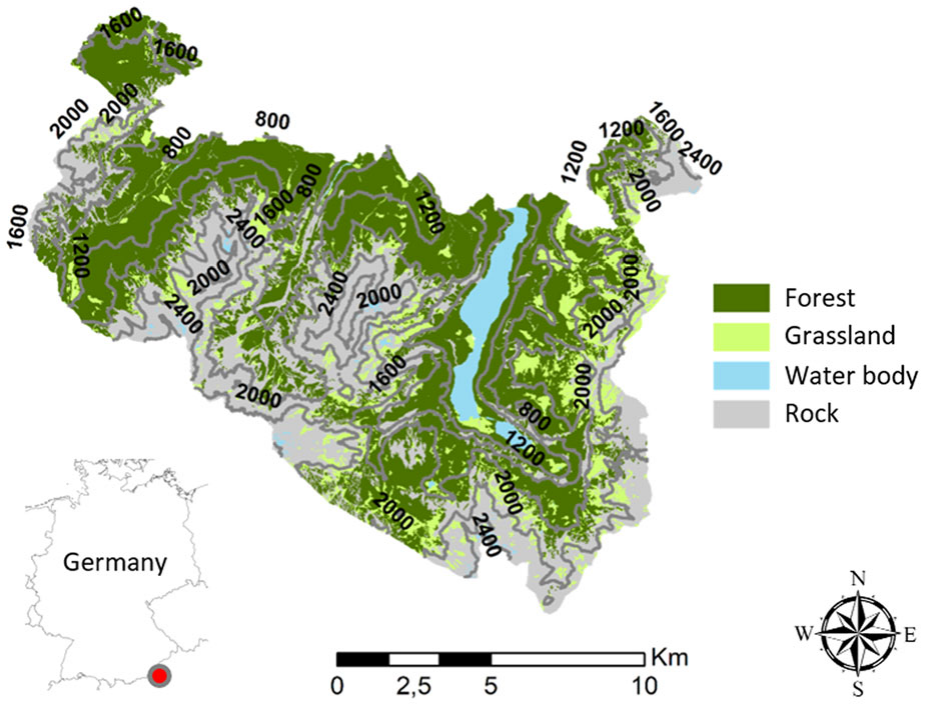
Berchtesgaden National Park. The red dot shows the position of the park in southeastern Germany. Isolines indicate elevation above sea level, colors show major land cover types.

**Figure 2:**
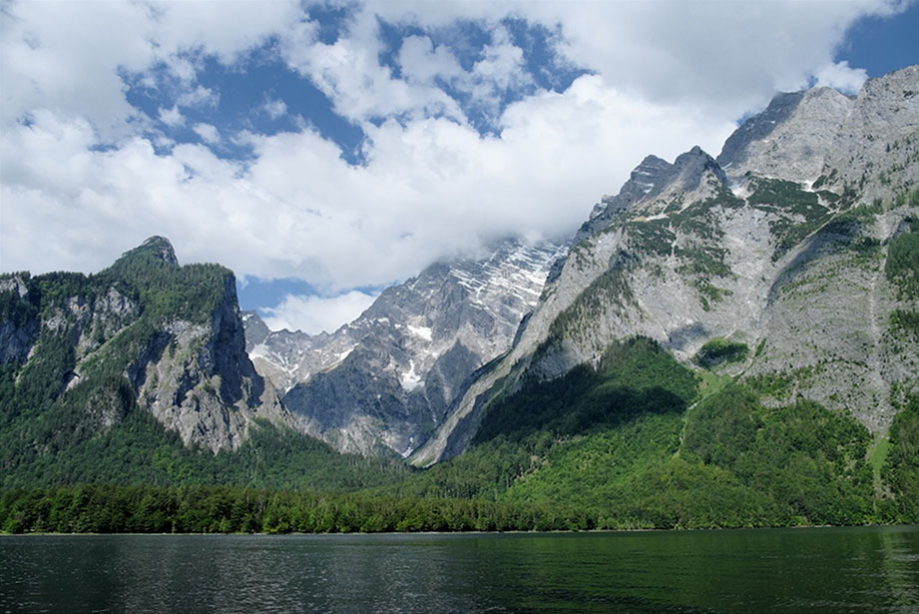
View from the lowest point on the landscape (lake Königssee, 603 m asl) toward the highest point (mount Watzmann, 2713 m asl, here hidden in the clouds), with the timber line at approximately 1700 m asl.

We investigated changes in forest ecosystems at BGNP in southeastern Germany (Figure 1). The landscape is characterized by high topographic complexity with elevations ranging from 603 m (lake Königssee) to 2713 m asl (mount Watzmann) (Figure 2). On average, the timber line is at approximately 1700 m asl. Forests cover a net area of 8645 ha of the 20,808 ha BGNP. In line with IUCN criteria, 75% of BGNP is strictly protected. With regard to forest area, interventions ceased entirely on 61.5%, whereas restoration management is conducted on the remaining 38.5%. Restoration management mainly consists of enrichment planting to facilitate a transition to natural species assemblages and bark beetle management along a buffer strip with neighboring commercial forests. Shallow Rendzic soil types and Cambisols of low to intermediate depth over calcareous bedrock are widespread across the landscape. At low elevations, the potential natural vegetation is dominated by European beech (*Fagus sylvatica* [L.]). Mixed forests consisting of Norway spruce, European beech, and silver fir (*Abies alba* [Mill.]) typically prevail in the montane elevation belt (approximately between 800 and 1400 m asl). With increasing elevation, Norway spruce gains dominance. The subalpine elevation belt (from 1400 m to the timber line) is characterized by Norway spruce forests as well as European larch—Swiss stone pine (*Pinus cembra* [L.]) forests.

### Data

#### Indicators of Forest Change

We analyzed nine indicators quantifying the change in forest structure and composition at BGNP. Individual tree data was obtained from three forest inventories conducted 1983–1985 (average 1984), 1995–1997 (average 1996), and 2010–2012 (average 2011). Taking all inventories together, we analyzed 132,866 records of trees with a diameter at breast height (dbh) at least 6 cm at 3759 permanent sample plots with a size of 500 m^2^. Plots were systematically distributed across the landscape on a 200 × 100 m grid and were permanently marked with a magnet hidden in the soil. For details on field methods, we refer to Becker (2012). We used individual tree data to derive plot-level aggregates of four indicators of stand structure and five indicators of tree species composition. Forest structure indicators were basal area (m^2^ ha^−1^), stand density (n ha^−1^), the number of large trees (dbh > 50 cm) per ha, and the variation in dbh, expressed as the standard deviation weighted by the stand density each tree represents per ha. Indicators of tree species composition were the proportion of conifers, the share of early seral species, late seral species, and rare species (all expressed relative to total basal area), and the effective tree species number calculated as the exponent of the Shannon Index (Hill number with q = 1). The classification of species in early-seral, late-seral, and rare species is given in Supplementary Table S1. The development of all indicators over time was expressed as average annual change within the two periods between the three inventories.

#### Potential Drivers of Forest Change

We considered 30 potential drivers of change in forest structure and composition, with drivers categorized into the four broad groups legacy, disturbance, climate, and site (Table 1). The pace of forest change is partly determined by the legacies of the past, providing the foundation for future development (Keeton and Franklin 2005). We included four indicators characterizing legacies of past forest development, whereof one was forest developmental stage (that is, gap/regeneration, establishment, early optimum, mid optimum, late optimum, plenter, terminal, decay) following the classification of Zenner and others (2016). Furthermore, we considered total basal area and stand age, retrieved directly from inventory data, as metric indicators of forest development state. In the inventory, the age of individual trees was derived by coring if no information was available for an individual from previous inventory periods. Young conifers were aged by counting whorls. Lastly, as BGNP was considerably influenced by humans in the past, we included an indicator quantifying past land-use in our analysis. Specifically, we derived the relative divergence between the tree species dominating the potential natural vegetation (PNV, derived from a wall-to-wall map for BGNP (Konnert 2004)) and the current species composition. To that end, we assumed that the dominant species of the PNV taken together account for at least 50% of stand basal area, and derived the relative difference between the observed share of the respective species and the PNV expectation. Taken together, these four indicators characterize the legacies of forest development over the past decades to centuries.

**Table 1:** Potential Drivers of Structural and Compositional Forest Change

Category	Attribute	Description	Unit	1984	1996
Legacies	Development stage	Eight development stages (gap/regeneration, establishment, early optimum, mid optimum, late optimum, plenter, terminal, decay) as defined in (Zenner and others 2016)	cat	–	–
	Total basal area	Sum of the stand basal area	m^2^ ha^−1^	23.3 (16.0)	27.4 (17.9)
	Stand age	Dominant (90th percentile) tree age	years	173.9 (82.5)	182.4 (83.5)
	PNV divergence	Divergence from expected potential natural vegetation	%	22.2 (39.6)	21.4 (39.1)

Category	Attribute	Description	Unit	1984–1996	1996–2011
Disturbance	Management zone	Strict protection zone or management zone	cat	–	–
	Disturbance occurrence	Natural disturbances and/or sanitary logging affecting a plot during the period	cat	–	–
Climate	Tmean winter	Average daily winter (DJF) temperature anomaly	°C	0.0 (0.0)	0.0 (0.0)
	Psum winter	Daily winter (DJF) precipitation anomaly	mm	7.6 (1.1)	− 17.0 (1.4)
	Rad winter	Average daily winter (DJF) solar radiation anomaly	MJ m^2^	− 0.1 (0.1)	0.1 (0.0)
	Tmean spring	Average daily spring (MAM) temperature anomaly	°C	− 0.3 (0.0)	0.2 (0.0)
	Psum spring	Daily spring (MAM) precipitation anomaly	mm	− 6.1 (1.5)	5.2 (0.8)
	Rad spring	Average daily spring (MAM) solar radiation anomaly	MJ m^2^	0.1 (0.1)	− 0.1 (0.1)
	Tmean summer	Average daily summer (JJA) temperature anomaly	°C	− 0.4 (0.0)	0.2 (< 0.0)
	Psum summer	Daily summer (JJA) precipitation anomaly	mm	1.7 (1.6)	3.0 (0.9)
	Rad summer	Average daily summer solar (JJA) radiation anomaly	MJ m^2^	0.0 (0.1)	0.0 (0.1)
	Tmean autumn	Average daily autumn (SON) temperature anomaly	°C	0.0 (0.0)	− 0.1 (0.0)
	Psum autumn	Daily autumn (SON) precipitation anomaly	mm	− 9.0 (1.8)	24.1 (1.8)
	Rad autumn	Average daily autumn (SON) solar radiation anomaly	MJ m^2^	− 0.1 (0.1)	0.0 (0.1)
	Tmean annual	Average daily temperature anomaly	°C	− 0.1 (0.0)	0.1 (0.0)
	Psum annual	Daily precipitation anomaly	mm	− 5.7 (2.8)	15.3 (0.9)
	Rad annual	Average daily solar radiation anomaly	MJ m^2^	0.0 (0.0)	0.0 (0.0)
Site	Elevation	Elevation above sea level	m	1263 (315)	1263 (315)
	Aspect	Cardinal direction of the plot	cat	–	–
	Roughness	Difference between the maximum and the minimum elevation of a cell and its eight surrounding cells	m	14.6 (8.7)	14.6 (8.7)
	TRI	Terrain Ruggedness Index: Mean of the absolute differences between the elevation of a cell and the elevation of its eight surrounding cells	m	4.4 (2.6)	4.4 (2.6)
	TPI	Topographic Position Index: Difference between the elevation of a cell and the mean elevation of its eight surrounding cells	m	0.0 (1.4)	0.0 (1.4)
	Sand	Sand fraction (soil texture)	%	45.3 (13.1)	45.3 (13.1)
	Clay	Clay fraction (soil texture)	%	20.4 (6.6)	20.4 (6.6)
	Soil depth	Effective soil depth available for rooting	cm	33.1 (18.6)	33.1 (18.6)
	Soil type	Soils grouped into the four most common soil types (rendzina soils, poor brown soils, rich brown soils, gleyic soils) found at BGNP	cat	–	–

Presented are means and standard deviations (in parentheses) for continuous variables in 1984 and 1996 (legacies) as well as in the two periods 1984–1996 and 1996–2011 (disturbance, climate, and site) across all 3759 inventory plots. PNV = potential natural vegetation. Roughness, TRI and TPI are calculated based on 10 m cells.

Disturbances may catalyze changes in forest ecosystems (Thom and others 2017b) and alter forest development pathways (Meigs and others 2017). Here, we tested the effect of two indicators of human and natural disturbance on observed forest change. First, we considered whether disturbances occurred in the study period, as identified from Landsat data at 30 m resolution (Senf and others 2017). Second, we considered whether a plot is in the strict protection zone of the national park (that is, only influenced by natural disturbances) or whether it is situated in the management zone (that is, subject to limited human disturbances, such as sanitation logging to halt bark beetle spread). The respective information was retrieved directly from inventory data.

Climate change can alter tree species composition and forest structure (Albrich and others 2020). We used daily high resolution (100 × 100 m) climate data that was dynamically generated with the Water Balance Simulation Model (WaSiM) (Schulla 2015) to derive average temperature and radiation as well as precipitation sums. WaSiM simulations were driven with time-series data of 35 meteorological stations (20 automatic and 15 mechanical stations) distributed across BGNP (14 stations) or located within close proximity (21 stations, Thom and others, in prep.). In addition to annual means, we calculated values for the four meteorological seasons. We derived differences between each period (that is, 1984–1996 and 1996–2011) and the average across all years (that is, 1984–2011) to characterize climate anomalies. In effect, climate anomalies were thus defined as the average climate for each inventory period subtracted by the climate of 1984–2011.

Site conditions constitute important environmental filters for forest composition and structure (Cui and Zheng 2016). We derived five topographic variables from a digital elevation model (DEM) at 10 m horizontal resolution. We computed elevation, aspect, terrain roughness (that is, the difference between minimum and maximum elevation of a cell and its eight surrounding cells), the Terrain Ruggedness Index (TRI, the mean of the absolute differences in elevation between a cell and its eight surrounding cells), and the Topographic Position Index (TPI, the difference in elevation of a cell and the average of its eight surrounding cells) (Wilson and others 2007). In addition, we considered four indicators describing soil conditions, including sand and clay fractions, effective soil depth and soil type (Konnert 2004).

### Analyses

#### Spatial Patterns

To address our first research question, we investigated spatial patterns of forest change over the 28-year period between 1984 and 2011. First, we derived annualized differences in each structural and compositional indicator between the years 1984 and 2011 for each plot. We divided these differences by the observed plot-level maximum, standardizing the maximum change of each indicator to 1. Subsequently, we analyzed the spatial autocorrelation of standardized changes for each indicator by means of semi-variograms. To that end, we derived the best fitting model among five model families (Spherical, Exponential, Gaussian, Matérn, Stein) using an automatic kappa value selection (Pebesma 2018). Final models were used to spatially interpolate changes across BGNP on a 100 m resolution by means of ordinary kriging which allows the integration of an autocorrelative structure. In addition, using the same approach, we derived spatial patterns of change for each observation period to enable a comparison of spatial patterns between them. In particular, we computed the root mean square error (RMSE) indicating the similarity between patterns as well as the congruence of trends (that is, a persistent positive or negative trend) between patterns across all pixels. To visualize spatial hot spots of forest change, we summed standardized changes between 1984 and 2011 of all indicators representing forest structure and composition, respectively, and divided by the number of variables to scale the maximum standardized change rate to 1.

#### Forest Change Rate

Subsequently, we investigated whether the rate of forest change remained stable over time, or whether an acceleration or deceleration of forest change could be detected. First, we derived average annual changes of each indicator within each observation period across all inventory plots. Next, we computed the relative annual change of each indicator compared to the first inventory (1984). Moreover, we subtracted the annual changes of each indicator in the period 1984–1996 from the annual changes of the period 1996–2011 to derive absolute changes between both periods. Positive values indicate an acceleration, negative values a deceleration, and values close to zero suggest constant change over time.

#### Drivers

To address our second research question, we quantified the effect of variables related to legacies, disturbance, climate, and site on forest change. We used boosted regression tree (BRT) models to analyze the influence of 30 covariates on nine response variables of structural and compositional change in both inventory periods. BRT models are composed of decision tree ensembles (Elith and others 2008). Covariates are added to a decision tree sequentially, aiming to explain the residual error of previously added covariates. BRT models account for non-linear effects and interactions, have a high predictive accuracy, and perform well if collinearity is high among covariates (Dormann and others 2013), as can be expected for our set of covariates. Moreover, BRT models provide a direct measurement of variable importance based on the number of times a variable is selected for splitting trees, weighted by the squared model improvement resulting from the splits (Elith and others 2008).

We performed a backward elimination of covariates based on variable importance obtained from tenfold cross-validation. As there is no widely accepted stopping criteria (such as AIC) for a BRT backward model selection, we calculated the RMSE of all candidate models resulting from the sequential elimination of covariates, and selected the model with the lowest error. As a visual inspection indicated approximately normal distribution of the data (Figure S1), we obtained the relative influence of covariates on response variables from the final models based on their marginal effects, and weighted them by the variance explained of the cross-validated test data to determine their combined impacts on forest change. If a variable was omitted during backward selection, its relative influence was set to zero. Final models were tested for residual spatial autocorrelation. Moran’s I statistic ranged from − 0.027 to 0.027, indicating that spatial autocorrelation did not impair final models.

For all analyses, we employed the R language and environment for statistical computing (R Development Core Team 2019). In particular, we used the packages tidyverse (Wickham 2019a), rgdal (Bivand and others 2018), spatialEco (Evans and others 2020), and RODBC (Ripley and Lapsley 2020) for data organization; gstat (Pebesma 2018) to fit variogram models and kriging; dismo (Hijmans and others 2017) for BRT models and their evaluation; spdep (Bivand and others 2019) to test spatial autocorrelation of BRT models; as well as ggplot2 (Wickham 2019b), ggmosaic (Jeppson and others 2018), and raster (Hijmans 2018) for visualizations.

## Results

### Spatial Patterns of Forest Change

All indicators of forest structure were on average higher in 2011 compared to 1984 (Figure S2–S5). Across the landscape, basal area increased by 0.343 m^2^ ha^−1^ y^−1^, stand density by 3.5 trees ha^−1^ y^−1^, large tree density by 0.6 trees ha^−1^ y^−1^, and SD DBH by 0.071 cm y^−1^. Changes in forest composition were less uniform across indicators (Figure S6–S10): The share of rare and late seral species as well as the effective tree species number increased (+ 0.007,  + 0.006, and + 0.003% y^−1^, respectively). In contrast, the share of conifers and early seral species decreased (− 0.014, and − 0.030% y^−1^, respectively).

**Figure 3:**
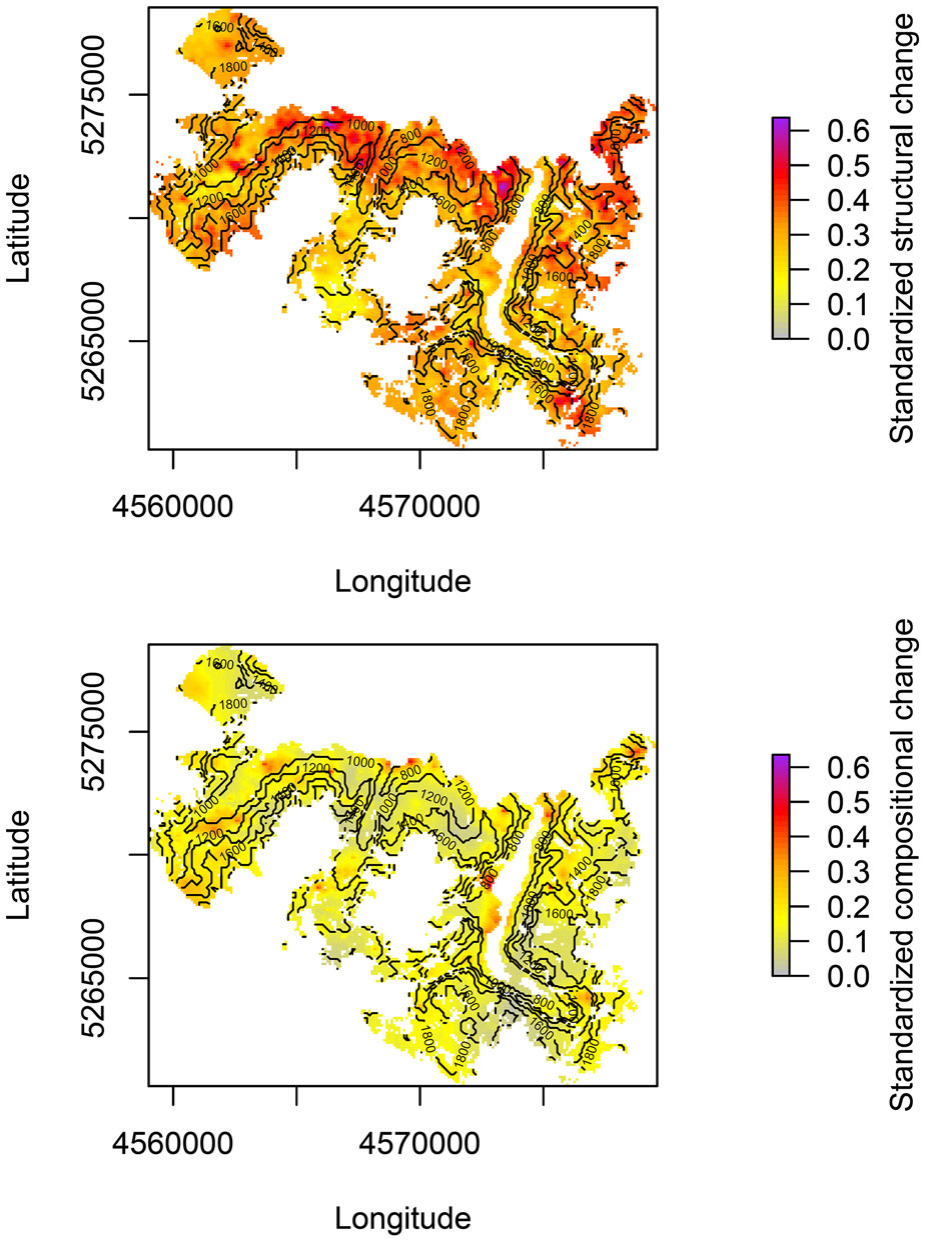
Changes in forest structure (top) and composition (bottom) at Berchtesgaden National Park between 1984 and 2011. Annual changes were standardized to the maximum observed plot level change per indicator, and were averaged across all structural (n = 5) and compositional (n = 4) indicators, respectively. Data of 3759 inventory plots were spatially interpolated to a 100 m grid by means of kriging with spatial autocorrelation determined by semi-variogram models. Isolines indicate elevation asl.

Spatial patterns of structural change were considerably more distinct than changes in forest composition (Figure 3, Figure S11). Although changes in forest structure were concentrated on the northern parts of BGNP, changes in composition were more homogenous across the landscape. On average, standardized changes in forest structure (0.31) were more than twice as strong as changes in composition (0.14). Neither for changes in forest structure nor composition a general trend across elevation was detected. However, changes in individual indicators varied with elevation (Figure S2–S10, S12). In particular, standardized changes in basal area (+ 0.08), conifer share (+ 0.11), and effective species number (+ 0.05) were higher below 1400 m asl (transition between montane and subalpine forests) than above 1400 m asl (Figure S12). In contrast, standardized changes in SD DBH (that is, variation of dbh) (− 0.10), and late seral species share (− 0.07) were lower below 1400 m asl than at higher elevations. Spatial patterns of change exhibited some variation, but trends were similar across both observation periods for most indicators of forest change (Table 2, Figure S13–S23). The spatial congruence of trends in structural attributes was high (89.3–100.0%). Trend patterns of compositional indicators had considerably higher variation, with lowest congruence between both periods for changes in late seral species (32.8%) and highest congruence for changes in effective species number (91.6%).

**Table 2:** Differences in Absolute Annual Changes of Compositional and Structural Indicators in the Two Observation Periods

Category	Attribute	Description	Unit	Inventories			Similarity of spatial patterns	
				Annual change 1984–1996	Annual change 1996–2011	Difference in annual change	RMSE	Trend congruence (%)
Structure	Basal area	Basal area change	m^2^ ha^−1^	0.342 (0.309)	0.344 (0.683)	0.002 (0.690)	0.186	96.6
	Stand density	Number of trees ≥ 6 cm dbh	n ha^−1^	2.136 (20.671)	4.589 (22.766)	2.453 (30.533)	6.006	89.3
	Large trees	Number of trees > 50 cm dbh	n ha^−1^	0.575 (1.191)	0.614 (1.472)	0.039 (1.888)	0.723	95.0
	SD DBH	Standard deviation of dbh	cm	0.060 (0.150)	0.081 (0.237)	0.021 (0.292)	0.046	100.0
Composition	Conifer ratio	Basal area proportion of conifers	%	0.008 (0.256)	− 0.032 (0.541)	− 0.040 (0.577)	0.104	40.5
	Early seral species	Basal area proportion of early seral species	%	− 0.028 (0.405)	− 0.032 (0.664)	− 0.004 (0.737)	0.243	62.5
	Late seral species	Basal area proportion of late seral species	%	− 0.014 (0.212)	0.022 (0.531)	0.036 (0.555)	0.046	32.8
	Rare species	Basal area proportion of rare species	%	− 0.000 (0.220)	0.012 (0.409)	0.012 (0.468)	0.028	46.3
	Effective species number	Effective number of tree species based on the exponent of the Shannon-Index	n	0.002 (0.013)	0.004 (0.021)	0.002 (0.024)	0.004	91.6

Differences in changes between inventories are presented as averages and standard deviations (in parentheses). The similarity of spatial patterns (Figure S13–S23) is expressed as the root mean square error (RMSE) of the difference between patterns (100 m resolution), with low values indicating high similarity in the spatial patterns of change over time. Furthermore, the congruence of trends in annual changes indicating whether positive or negative trends persist at the same location throughout both observation periods (expressed in % of pixel on a 100 m resolution with congruent trends over both inventory periods). Inventories were conducted in 1984, 1996, and 2011 across 3759 permanent sample plots in Berchtesgaden National Park.

The spatial autocorrelation of forest change was generally weak, and more distinct for structural change than for compositional change (Figure S11). The semi-variance of basal area, stand density, the number of large trees per ha, and rare species decreased moderately within close proximity (with distances of 247, 475, 147, and 54 m at which the models flatten). The spatial dependence of change in other variables was negligible.

### Acceleration of Forest Change

**Figure 4:**
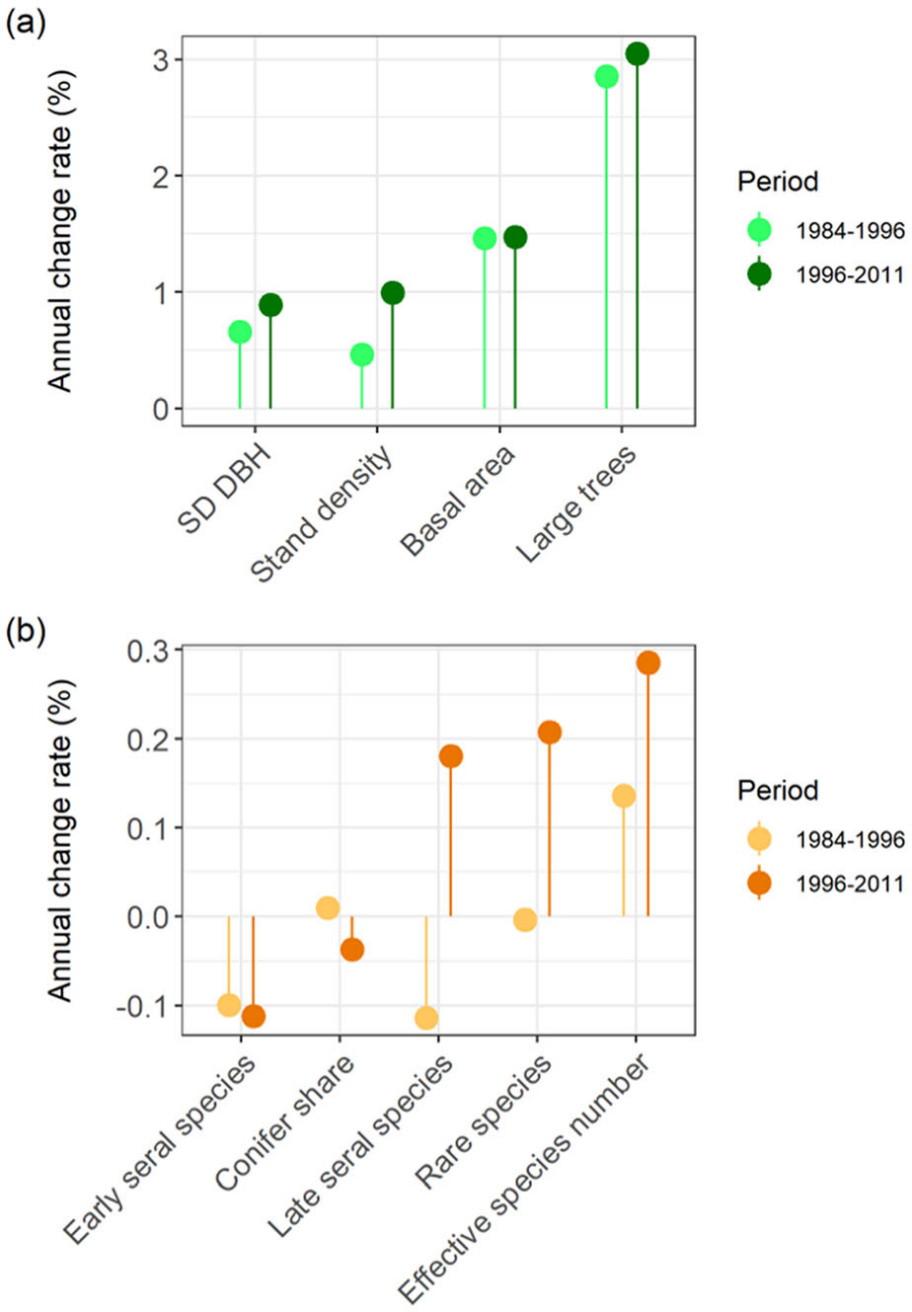
Temporal trends in forest change at Berchtesgaden National Park. Presented are annual changes in **a** forest structure and **b** composition over two inventory periods (1984–1996, 1996–2011) relative to the first inventory (1984). For a description of variables see Table 2. Please note the differently scaled y-axes of both panels.

Structural and compositional changes across BGNP accelerated over the study period (Figure 4, Table 2). In absolute terms (that is, disregarding directionality of change), all indicators changed more rapidly in the second period (1996–2011) compared to the first (1984–1996). All indicators of structural change increased in the second period compared to the first. Relative to the first inventory in 1984, the indicator changing most strongly throughout both periods across the landscape was the share of large trees (Figure 4). In general, change rates in forest structure were an order of magnitude stronger than those of compositional indicators. The increase in effective tree species number and the decrease in early seral species were accelerated from the first to the second period. Changes of other compositional indicators switched signs between both periods.

### Drivers of Forest Change

**Figure 5:**
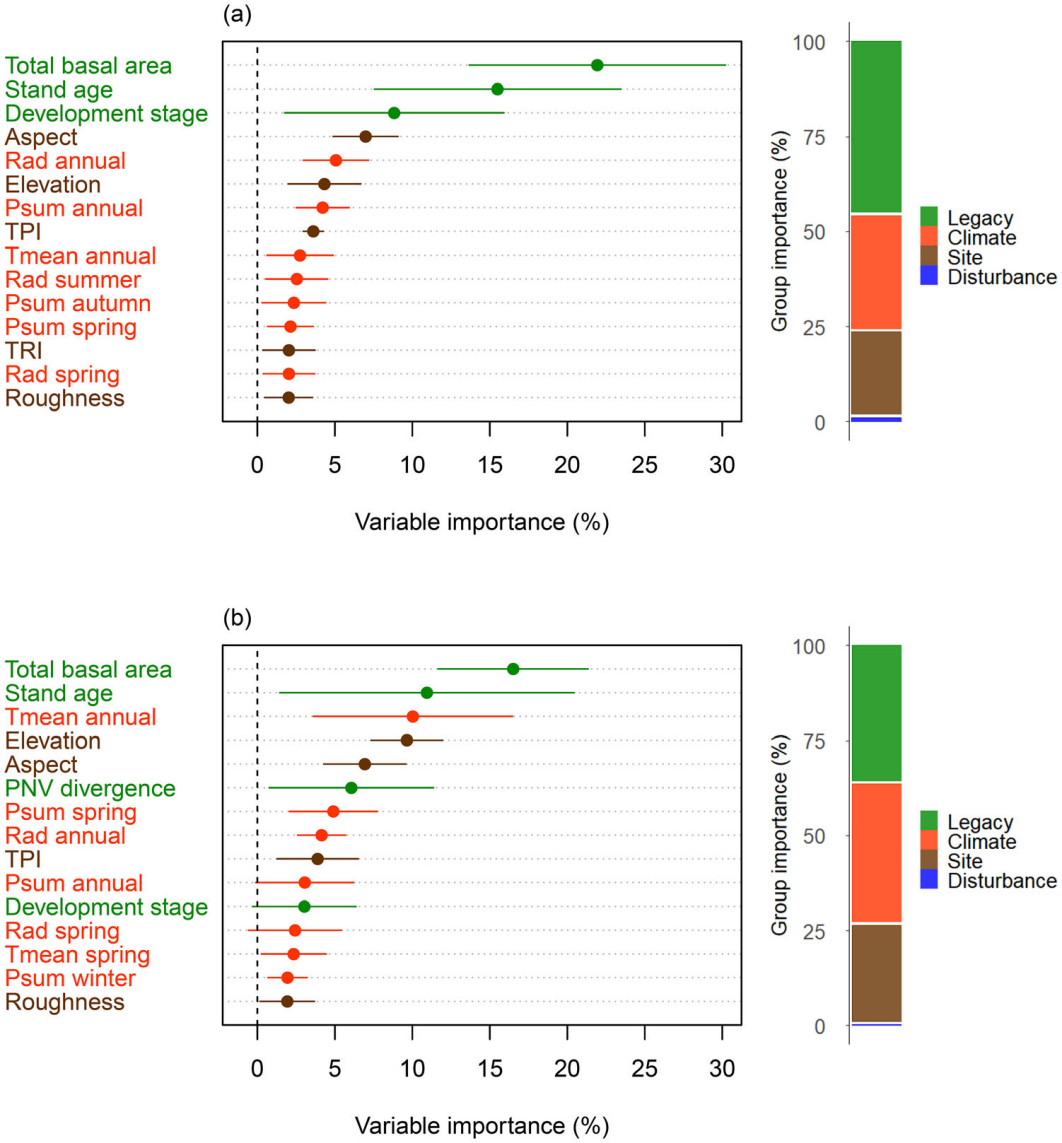
Relative importance of drivers and driver categories explaining forest change at Berchtesgaden National Park. Shown are the 15 most important **a** drivers of structural change, and **b** drivers of compositional change, respectively (for all drivers see Figure S32). Presented are weighted averages (dots) and confidence intervals (whiskers) for the relative importance of drivers explaining the combined changes in four structural and five compositional variables across two inventory periods (1984–1996, 1996–2011) based on BRT models. The relative importance of each driver was weighted by the variance explained in cross-validation. Bar charts summarize the relative importance of each driver category (across all 30 potential driver variables, see Table 1) in explaining structural and compositional changes.

Legacies and climate were the strongest drivers of forest change at BGNP (Figure 5; Figure S22–S32). BRTs explained 23.8% (SD ± 13.5%) of the variation in the data on average across all models based on the test data used for cross-validation. Models of structural change performed considerably better (34.6 ± 11.1%) than those modeling changes in composition (15.2 ± 7.8%). With a relative contribution of 46.3 and 36.5%, legacy variables were a major driver of structural and compositional changes, respectively. Also climate (30.5 and 37.2%, respectively) and site (22.2 and 26.1%, respectively) variables had a considerable influence on the observed changes in forest structure and composition. In contrast, disturbances exerted only a weak influence on forest change (1.0 and 0.2%, respectively). The most important individual covariates for changes in forest structure and composition were total basal area (21.9 and 16.5%, respectively), stand age (15.5 and 10.9%, respectively), aspect (7.0 and 6.9%, respectively), development stage (8.8 and 3.0%, respectively), and anomalies in average annual temperature (2.8 and 10.0%, respectively).

Overall, legacies and climate had opposing effects on the magnitude of forest change (Figure S22–S30). In particular, average annual temperature amplified changes (that is, an increase causes change rates to diverge from 0). For instance, increases in average annual temperature were negatively correlated with changes in conifer share (Figure S26), and positively associated with alterations in late seral species share (Figure S28). In contrast, high legacy levels (for example, development stages closer to old-growth conditions) dampened forest change, with an increase in indicators causing change rates to plateau close to 0. For instance, while total basal area and stand age were negatively associated with changes in stand density, the negative correlation vanished as total basal area and stand age increased (Figure S23). The same held true for the negative effect of total basal area on changes in SD DBH (Figure S25), early seral species share (Figure S27) and effective species number (Figure S30).

## Discussion

We studied the patterns and drivers of forest change in a mountain forest landscape based on a high-density forest plot network with three consecutive censuses over a period of 28 years. We show that the forests of BGNP are recovering from past land-use and are developing toward older, structurally and compositionally more complex forest ecosystems. In line with other observations studying systems developing toward old-growth conditions (Tyrrell and Crow 1994; Bauhus and others 2009; Meigs and others 2017; Urbano and Keeton 2017), we found that basal area, the number of large trees, the variation in tree dimensions, the share of rare and late seral species, and tree species diversity increased while the share of early seral species decreased. Interestingly, we also found an increase in stand density over time, despite the expectation that stand density decreases with forests age (Fischer and Fischer 2012; Mette and others 2013). This finding could be the result of incipient gap dynamics in older forests (Diaci and others 2012) in combination with small-scale disturbances affecting the landscape during the observed period (Senf and others 2017). It could also reflect warmer climate conditions, as tree establishment of mountain forests has been limited by short vegetation periods and heavy snow cover in the past (Johnson and Yeakley 2019). However, our analysis on stand density drivers does not support this notion, with stand density changing faster in less cold-limited low elevation parts of the landscape (Figure S23). Consistent with a fingerprint of climate warming (Parmesan and Yohe 2003)is the observed decrease in conifer share (Figure S26), as broadleaved species and, in particular, European beech are expected to gain dominance in the Northern Alps under continued climate change (Mette and others 2013; Thom and others 2017b). Although warming rates increase with elevation in the Alps (Gobiet and others 2014), we did not detect a general elevation trend throughout all indicators of forest change (Figure 3, Figure S21). In fact, changes in basal area (Figure S3), conifer share (Figure S6), and effective tree species number (Figure S10) were more pronounced at low elevations (Figure S12). The transition toward broadleaved species at low to mid-elevations might thus be a result of a legacy of historic forest management favoring Norway spruce in these parts of the landscape (see also Thom and others 2018). Furthermore, as historic environmental filters at high elevations limited tree species diversity and climate change-induced tree species migration is a very slow process in mountain forests (Thom and others 2017b), it is possible that changes in diversity and composition of high elevation forests lag behind low elevation forests as the availability of seeds limits compositional responses to novel climatic conditions.

Forest change has accelerated considerably over the 28-year study period at BGNP. Confirming our expectation, forest structure changed faster than forest composition (Figures 3, 4). Moreover, supporting our initial hypothesis, climate change was a main driver of forest change (Figure 5, Figure S22–S30). Interestingly, climate exerted a stronger influence on compositional changes compared to structural changes, with structure being more strongly driven by legacies of past forest development. We note that the difference in annual average temperature (the most influential climate variable in our analysis, Figure 5) between the two periods was 0.21°C (corresponding to a warming of 0.07°C per decade, Table 1). As a further acceleration of climate change is likely (Nazarenko and others 2015), we expect an amplification of changes at BGNP in the future.

We found that development toward old-growth conditions may compensate impacts of climate change on forest dynamics. This is in line with a recent study that provides evidence for a decrease in the climate sensitivity of forest ecosystems as forests age (Thom and others 2019). However, in the long term, it is also possible that climate change exceeds a tipping point after which old-growth forests exhibit higher climate sensitivity than younger forests. For instance, an intensification of droughts will more strongly affect older, larger trees (Stovall and others 2019). Hence, future drought disturbances may cause abrupt changes in the structure and composition of old-growth forests. The interactions between forest development and climate change thus warrant further analyses.

Spatial patterns and the directionality of change were more distinct for forest structure than composition (Figures 3, 4, Table 2, Figure S11). This finding on the spatial patterns of change is somewhat surprising, given that processes driving species change (for example, seed dispersal) are generally more strongly dependent on spatial proximity than processes determining forest structure (for example, tree growth and mortality) (Auffret and others 2017). Our findings suggest that the observed species changes are primarily the result of shifts in the competitive balance of already established species rather than the result of a large-scale migration of tree species (Tylianakis and others 2008). We hypothesized that disturbances are important catalysts of forest change, influencing its spatial patterns and directionality (Thom and others 2017b; Dietz and others 2020). However, we did not find strong support for this hypothesis in our data (Figure 5, Figure S32). In this context, important limitations of our study have to be considered. The natural disturbance indicator used here only captured the occurrence of disturbance but did not account for disturbance severity. Further uncertainties exist with regard to potential spatial mismatches in aligning the relatively coarse disturbance map (30 × 30 m) with inventory plot locations. The limited impact of disturbance on ecosystem dynamics found here is likely also related to the moderate disturbance activity at BGNP during the study period (Senf and others 2017). As climate change will amplify disturbance regimes (Seidl and others 2017; Thom and others 2017c), a stronger effect of natural disturbances on ecosystem dynamics can be expected in the future. With regard to our hypothesis on the buffering effect of topographic complexity on forest change (Albrich and others 2020) we did not find a clear signal in our data. Variables related to topography (elevation, aspect, TPI, TRI, roughness) were important factors modulating forest change (Figure 5, Figure S32). Yet the directionality of their effects could not be generalized from our results (Figure S22–S30). Future work should thus investigate the role of topography more closely, for example, explicitly searching for topographically determined refugia of forest change on the landscape (Loarie and others 2009).

We conclude that forests at BGNP are changing at an accelerating pace. The impacts of this acceleration remain widely unclear and should be the focus of future research. It, for instance, remains unresolved whether accelerated forest dynamics have positive (for example, as particularly species-rich development stages appear faster, Hilmers and others 2018) or negative (for example, because taxa are not able to keep up with the increasing pace of forest dynamics, Murray and others 2017) effects on biodiversity. Furthermore, whether a continued acceleration of forest change will eventually lead to disruptions as tipping points are crossed remains unclear (Turner and others 2020). We here show that the dynamics of unmanaged forests developing toward old-growth conditions can counteract climate-mediated acceleration to some degree (cf. Thom and others 2019). This suggests that protected areas such as national parks could become important refuges in landscapes that increasingly gravitate toward accelerated demographic processes (“faster in – faster out” dynamics). It furthermore underlines that natural forest dynamics provides important lessons for ecosystem management in the context of adapting to the impacts of global change.

## Supplementary Information

Below is the link to the electronic supplementary material.

Supplementary file1 (DOCX 20577 KB)
