# Supplementary material for: Accelerating Mountain Forest Dynamics in the Alps
Source: Ecosystems. 2021 Jul 28;25(3):603–17. doi: 10.1007/s10021-021-00674-0 (PMC9016046; doi:10.1007/s10021-021-00674-0)
Supplement: Supplementary file 1 — Supplementary file1 (DOCX 20577 KB) [file 10021_2021_674_MOESM1_ESM.docx]

**Supplement**

**Indicators of forest change**

Table S1: Classification of species in early-seral, late-seral, and rare.

| Classification | Criteria | Species |
| --- | --- | --- |
| Early-seral | Low maximum age; early culmination of height growth; low shade tolerance | *Alnus spp*., *Betula spp*., *Juniperus communis*, *Larix decidua*, *Malus spp*., *Pinus sylvestris*, *Populus spp*., *Prunus avium*, *Pyrus spp*., *Salix spp*., *Sorbus spp*. |
| Late-seral | High maximum age; late culmination of height growth; high shade tolerance | *Abies alba*, *Fagus sylvatica*, *Quercus spp*., *Pinus cembra*, *Taxus baccata* |
| Rare | Species that are below the analysis threshold for individual species in the German National Forest Inventory (<https://bwi.info>) and are not dominant species in major forest types of Berchtesgaden National Park | *Acer ssp.*, *Fraxinus ssp.*, *Pinus uncinata*, *Tilia spp*., *Ulmus spp*. |


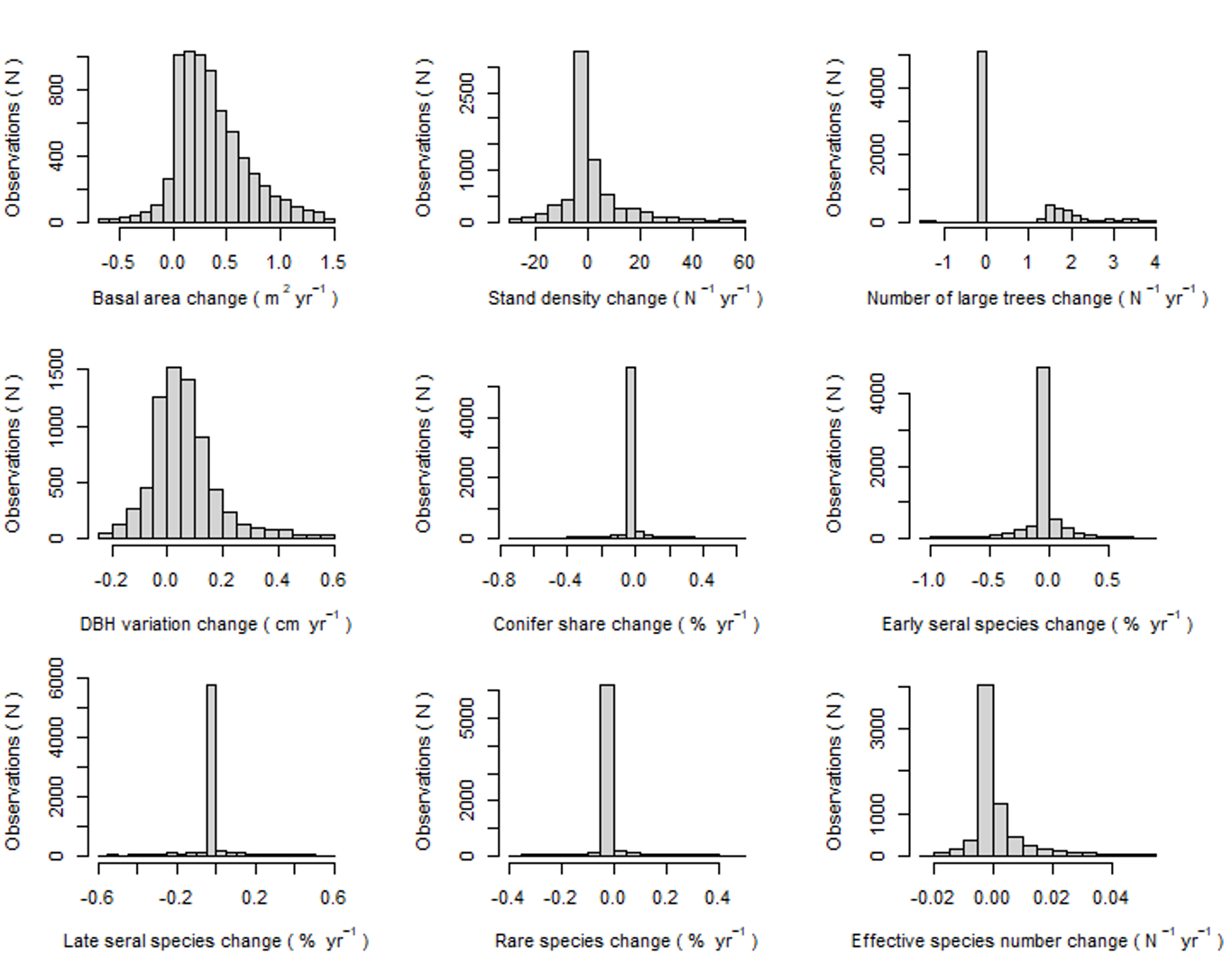


Figure S1: Data distribution of annualized absolute changes including both inventory periods (i.e., 1984 – 1996 and 1996 – 2011). x-axes were trimmed from each end by 1% of observations to omit outliers in the visualization.

**Spatial distribution of forest change**


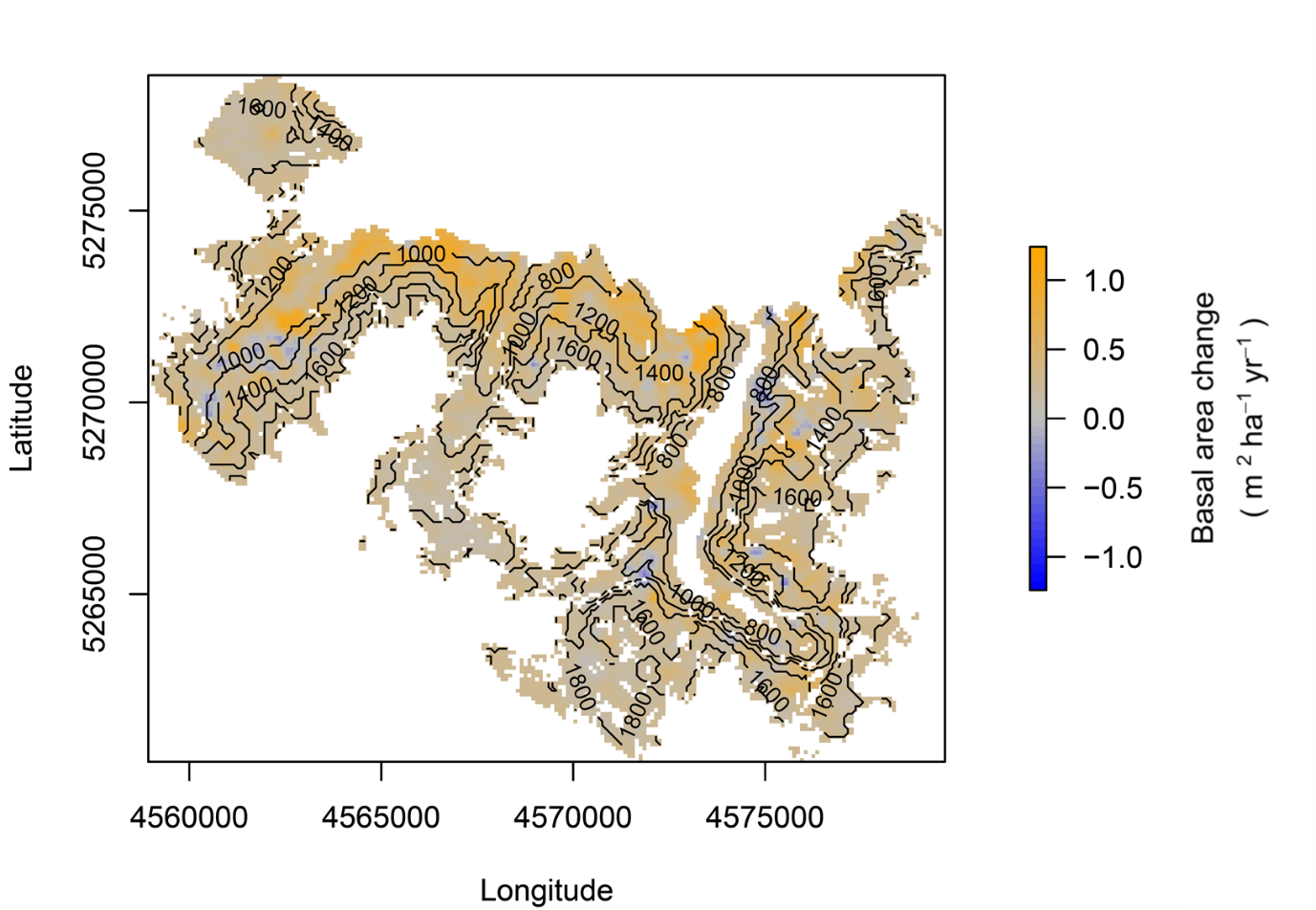


Figure S2: Spatial distribution of absolute annual changes in basal area between the year 1984 and 2011. Values of 3,759 inventory plots were spatially interpolated on a 100 m grid by means of kriging with spatial autocorrelation determined by semi-variogram models (Fig. S2). Isolines indicate elevation asl.


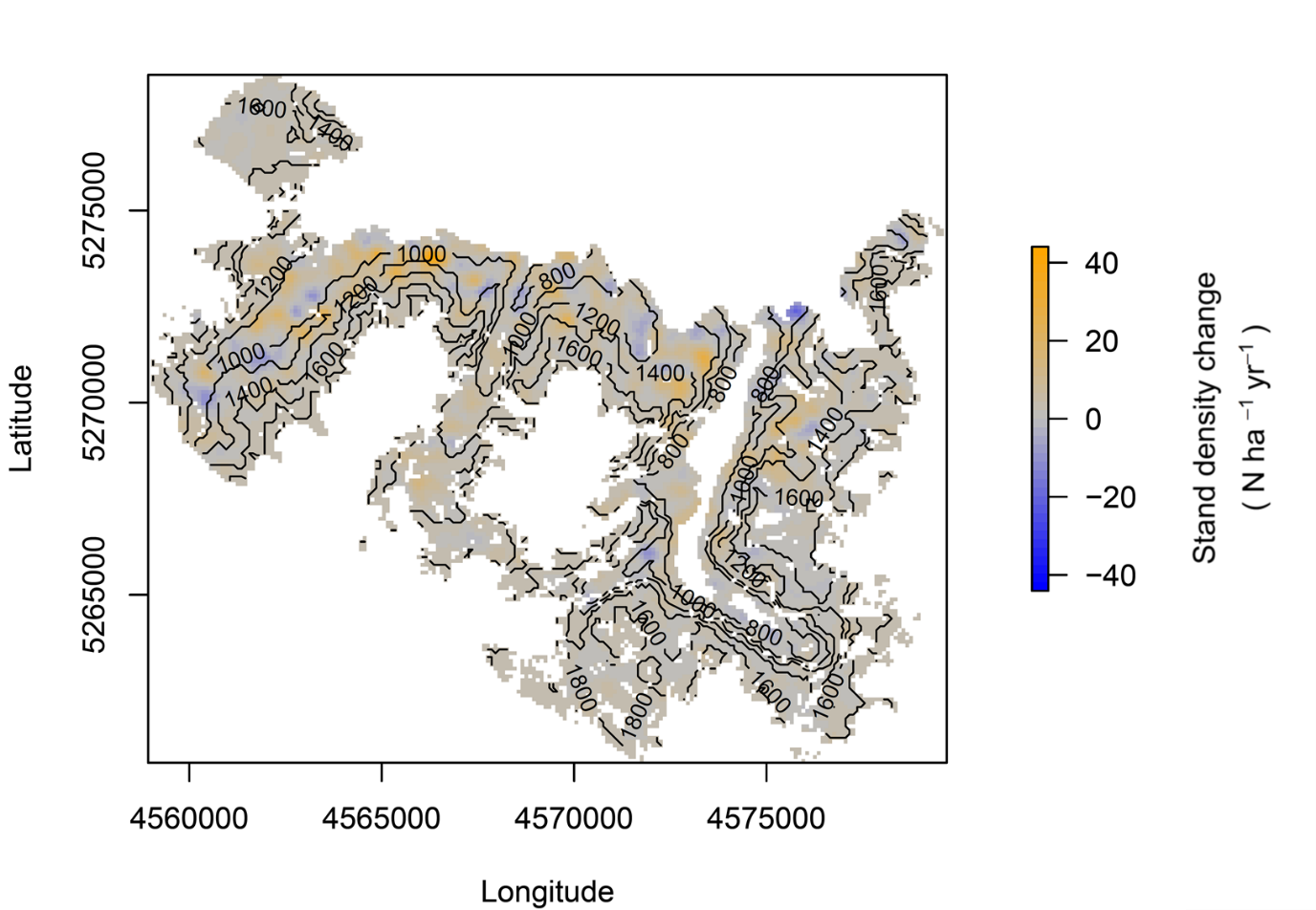


Figure S3: Spatial distribution of absolute annual changes in stand density between the year 1984 and 2011. Values of 3,759 inventory plots were spatially interpolated on a 100 m grid by means of kriging with spatial autocorrelation determined by semi-variogram models (Fig. S2). Isolines indicate elevation asl.


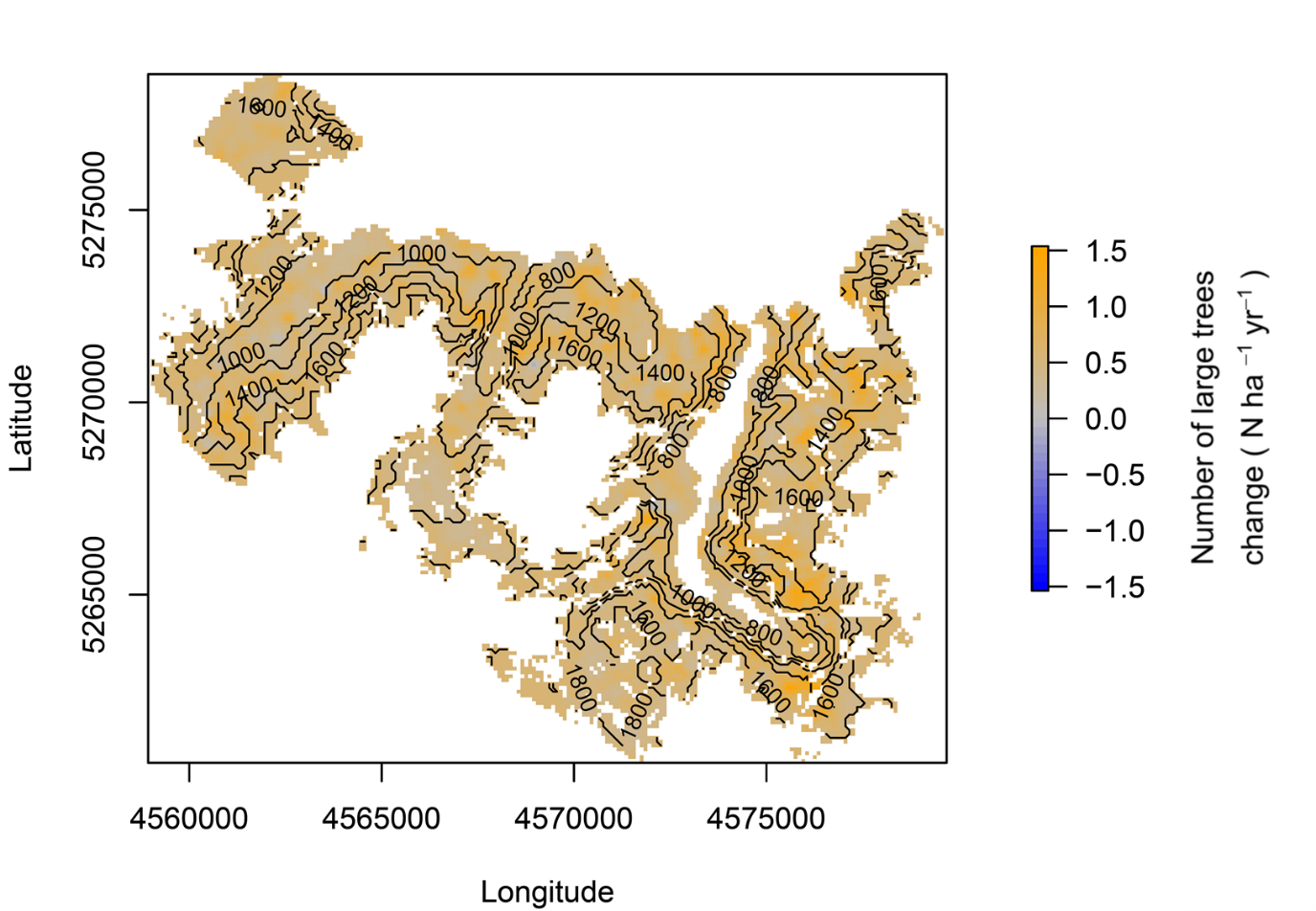


Figure S4: Spatial distribution of absolute annual changes in the number of large trees (DBH > 50 cm) per hectare between the year 1984 and 2011. Values of 3,759 inventory plots were spatially interpolated on a 100 m grid by means of kriging with spatial autocorrelation determined by semi-variogram models (Fig. S2). Isolines indicate elevation asl.


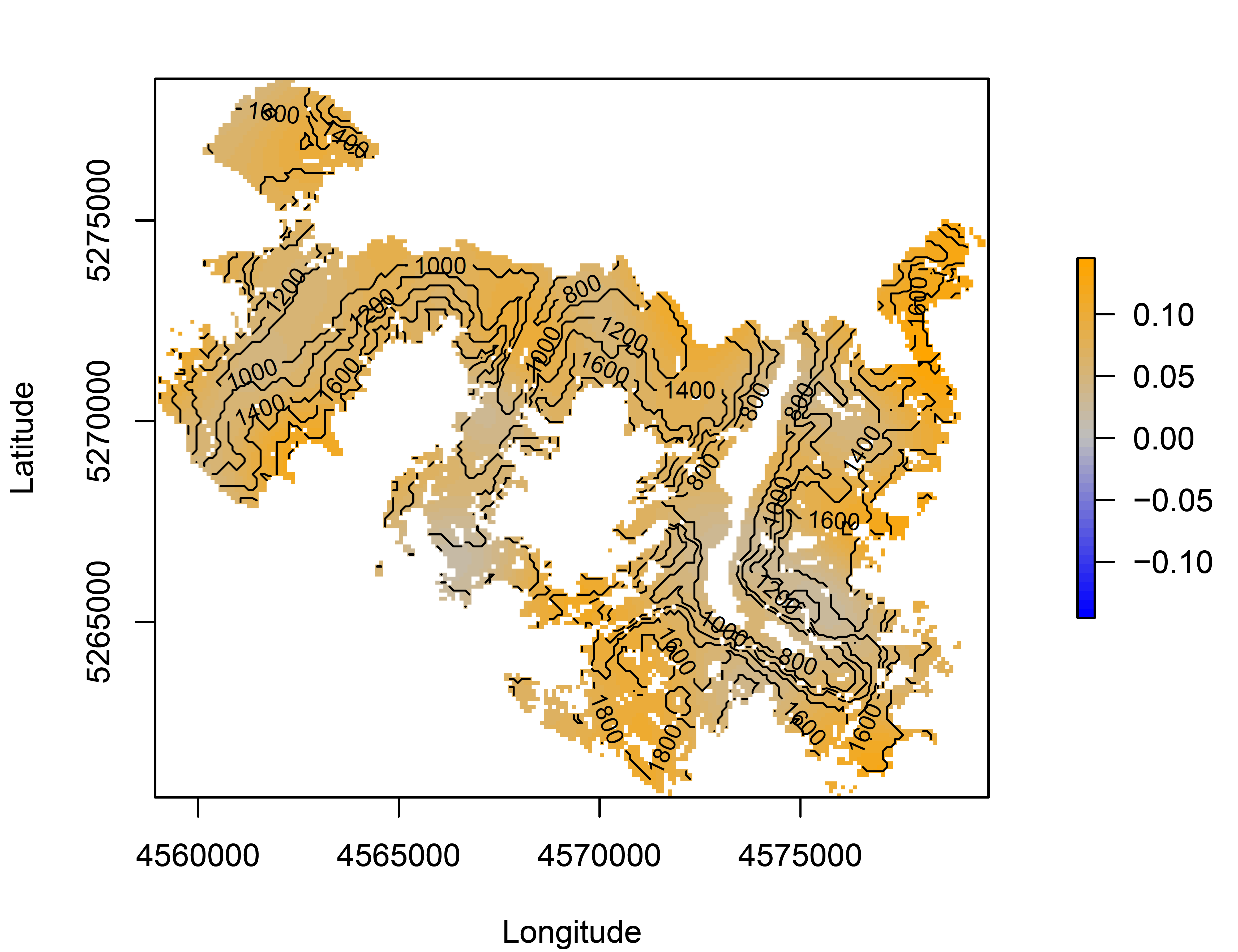


Figure S5: Spatial distribution of absolute annual changes in DBH variation (expressed as standard deviation of DBH) between the year 1984 and 2011. Values of 3,759 inventory plots were spatially interpolated on a 100 m grid by means of kriging with spatial autocorrelation determined by semi-variogram models (Fig. S2). Isolines indicate elevation asl.


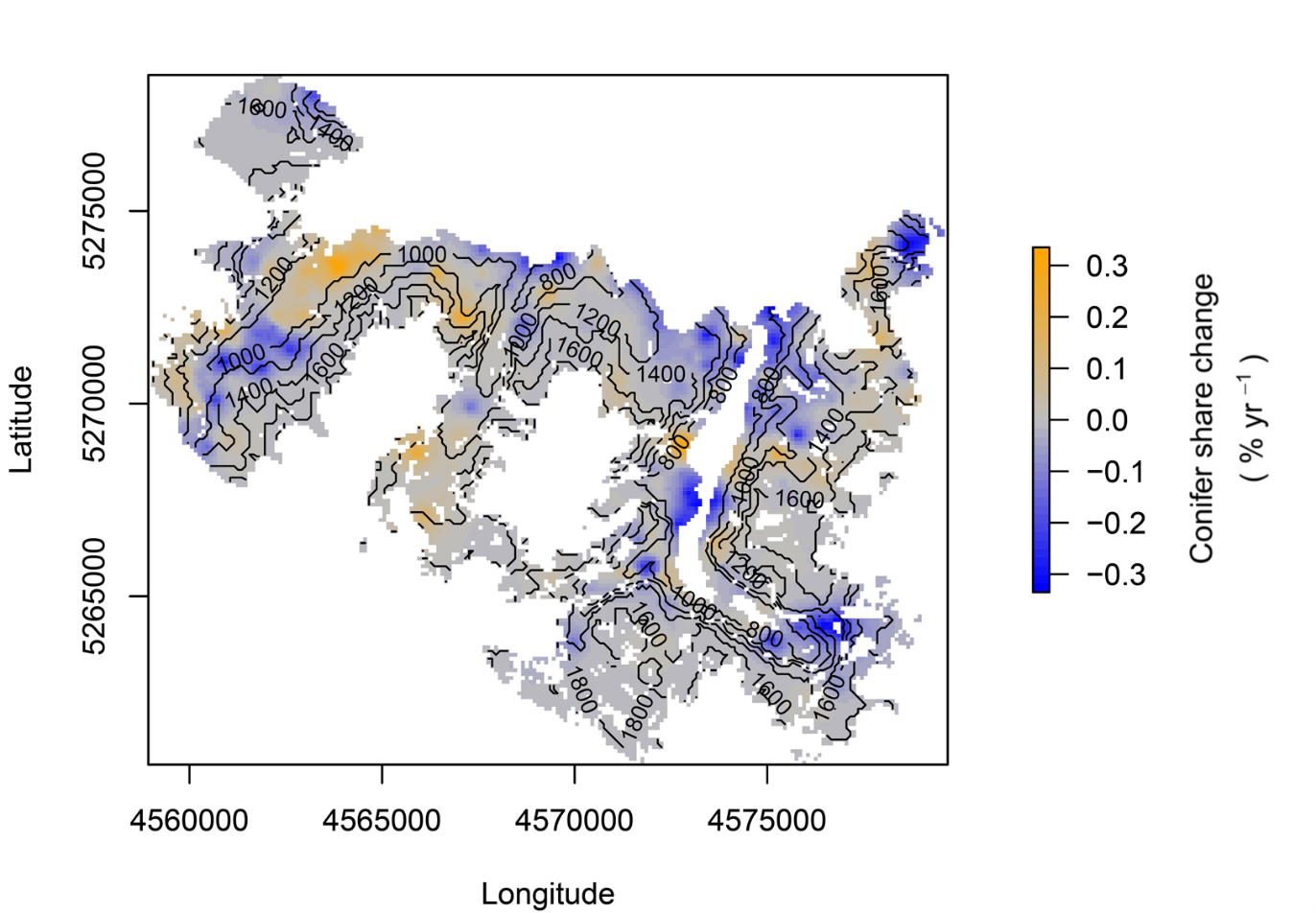


Figure S6: Spatial distribution of absolute annual changes in conifer share between the year 1984 and 2011. Values of 3,759 inventory plots were spatially interpolated on a 100 m grid by means of kriging with spatial autocorrelation determined by semi-variogram models (Fig. S2). Isolines indicate elevation asl.


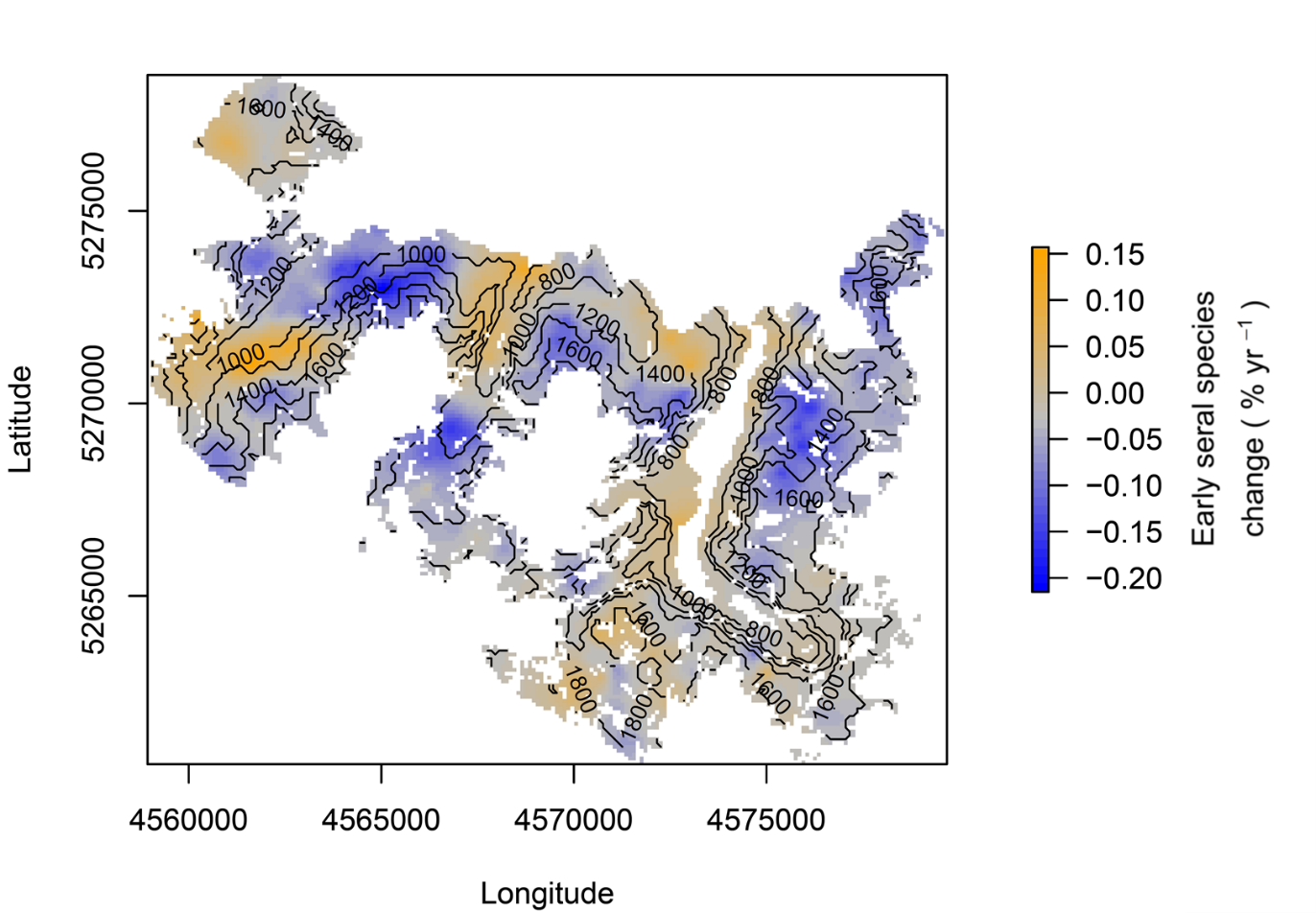


Figure S7: Spatial distribution of absolute annual changes in the share of early seral species between the year 1984 and 2011. Values of 3,759 inventory plots were spatially interpolated on a 100 m grid by means of kriging with spatial autocorrelation determined by semi-variogram models (Fig. S2). Isolines indicate elevation asl.


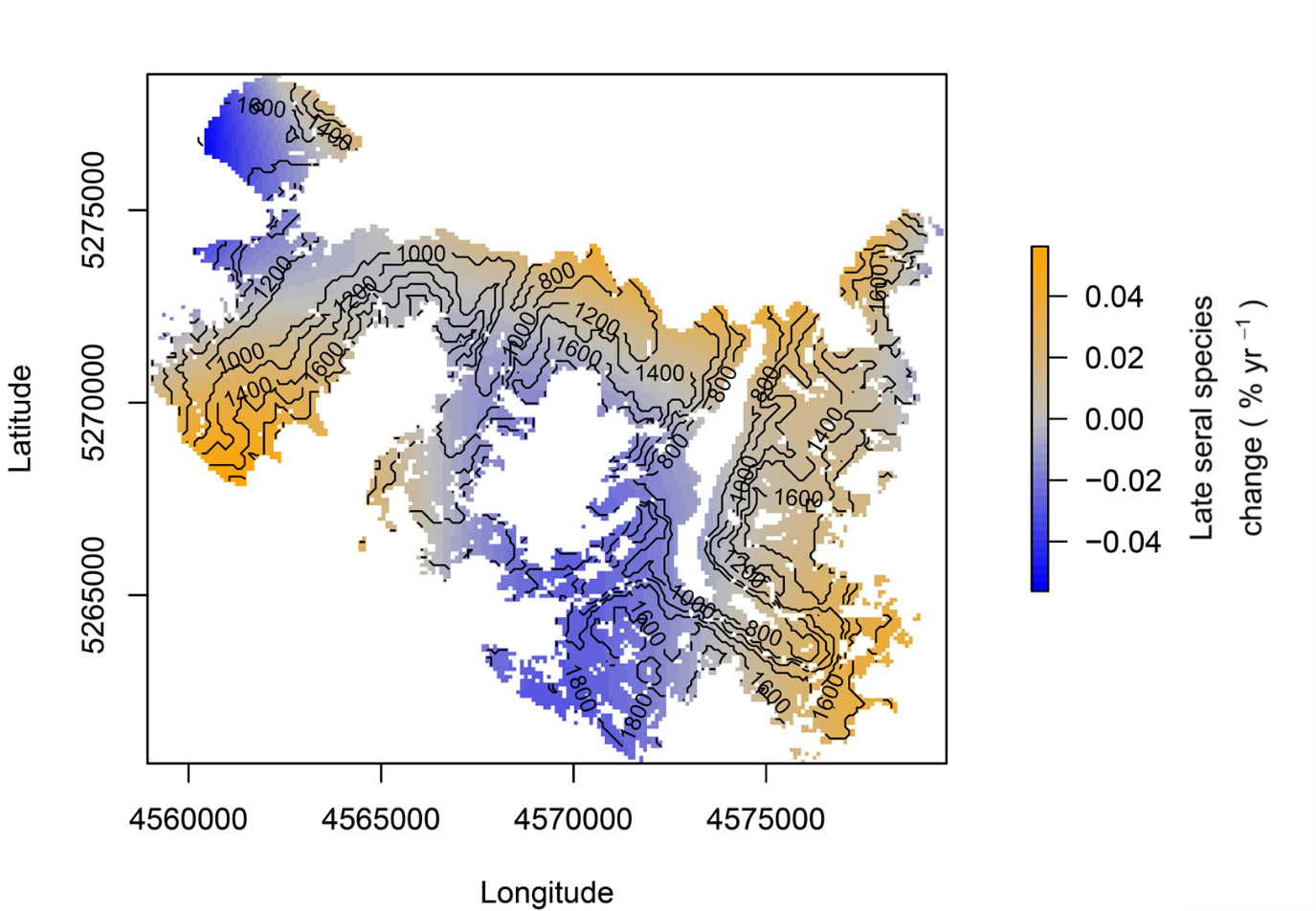


Figure S8: Spatial distribution of absolute annual changes in the share of late seral species between the year 1984 and 2011. Values of 3,759 inventory plots were spatially interpolated on a 100 m grid by means of kriging with spatial autocorrelation determined by semi-variogram models (Fig. S2). Isolines indicate elevation asl.


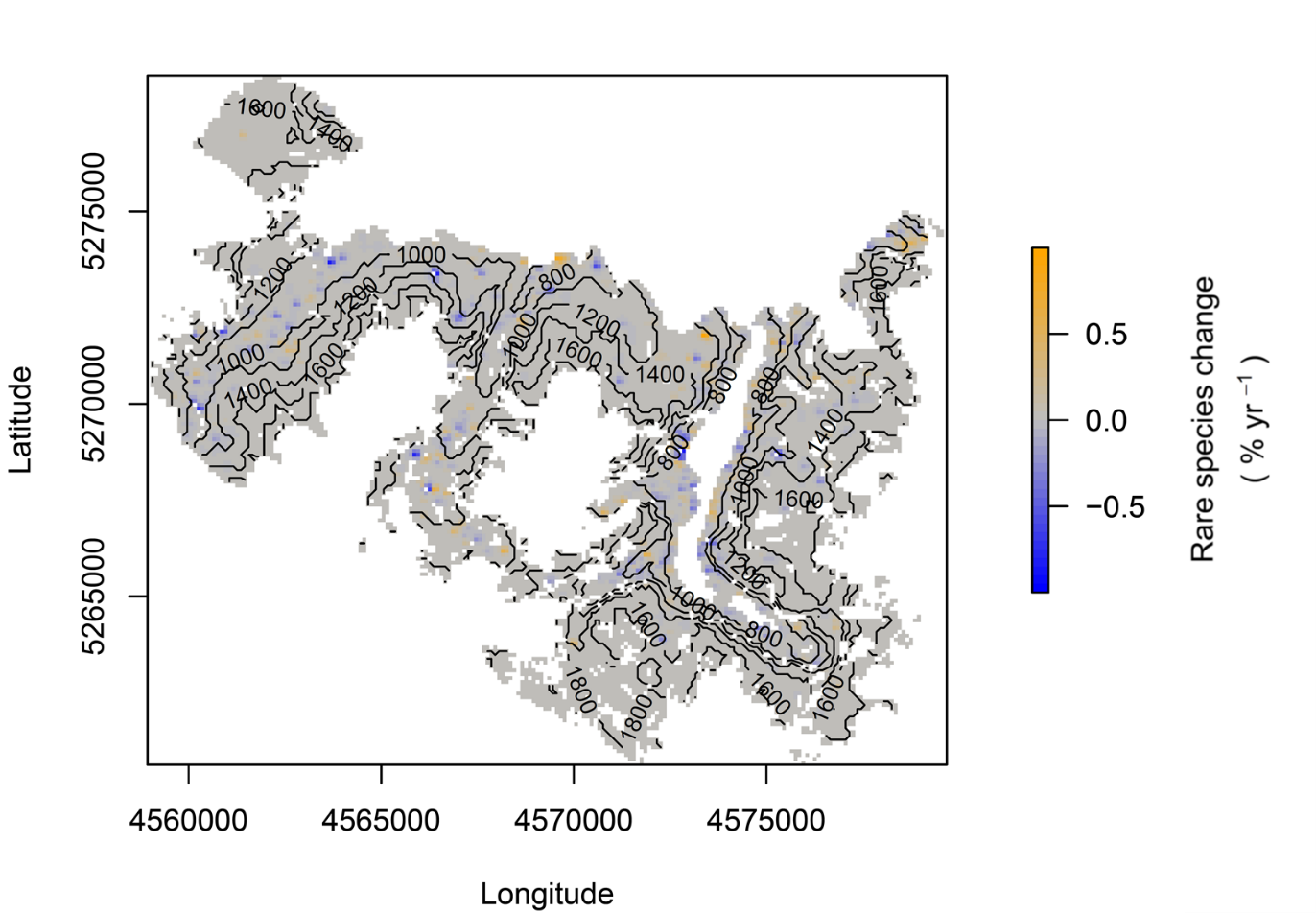


Figure S9: Spatial distribution of absolute annual changes in the share of rare species between the year 1984 and 2011. Values of 3,759 inventory plots were spatially interpolated on a 100 m grid by means of kriging with spatial autocorrelation determined by semi-variogram models (Fig. S2). Isolines indicate elevation asl.


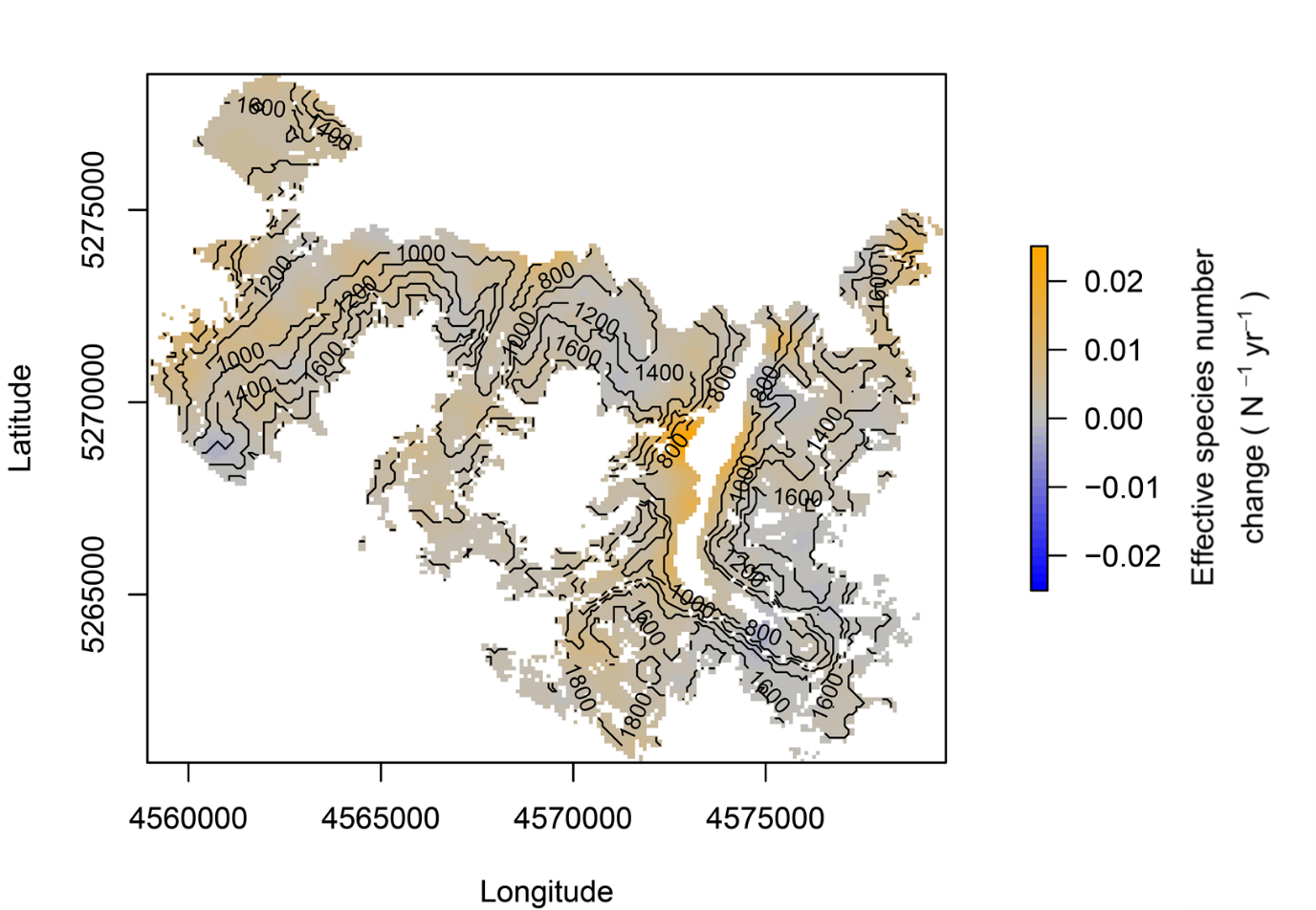


Figure S10: Spatial distribution of absolute annual changes in effective species number (expressed as the exponent of the Shannon-Index) between the year 1984 and 2011. Values of 3,759 inventory plots were spatially interpolated on a 100 m grid by means of kriging with spatial autocorrelation determined by semi-variogram models (Fig. S2). Isolines indicate elevation asl.


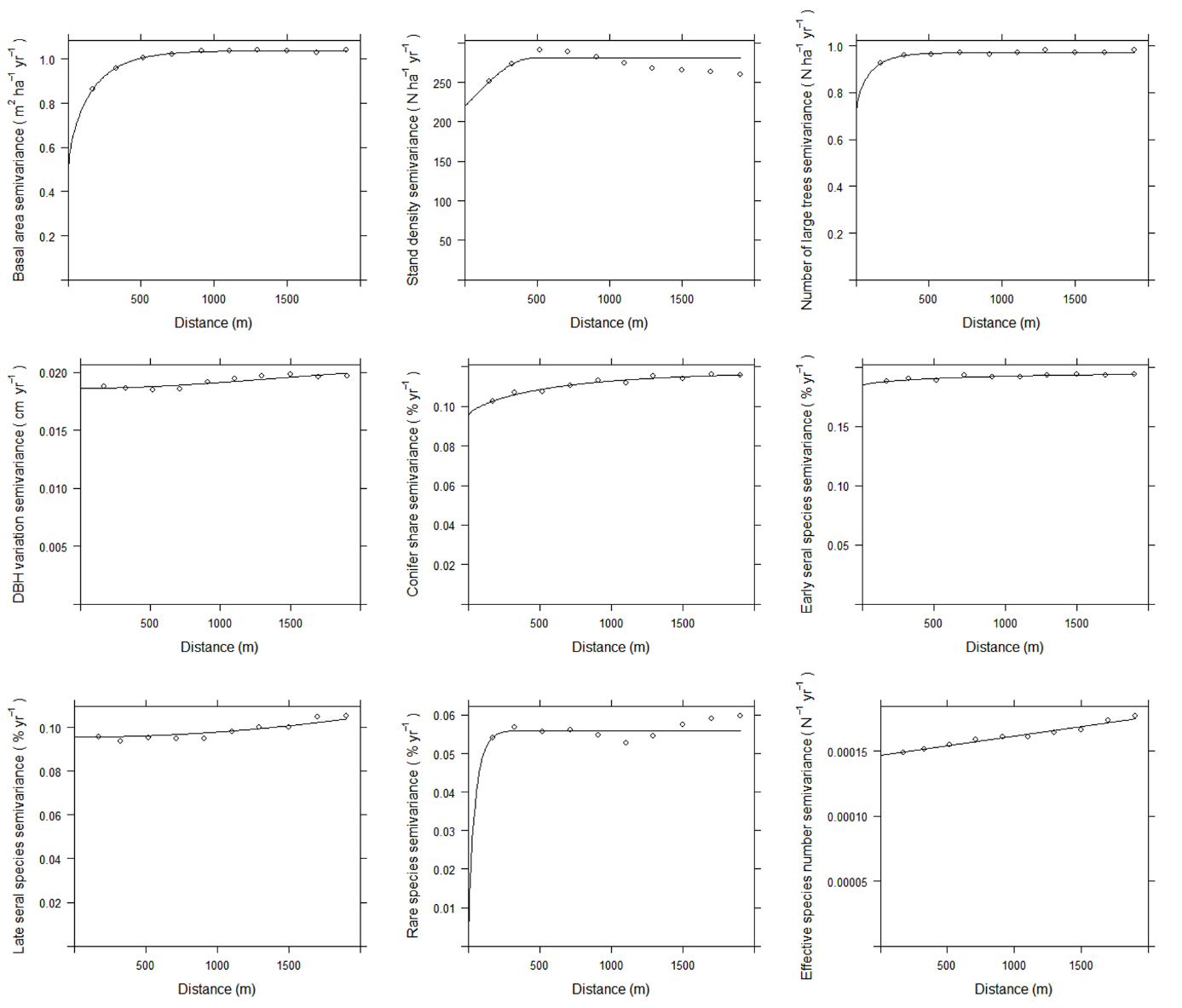


Figure S11: Semi-variograms of the spatial autocorrelation of annualized changes in forest structure and composition at BGNP across 3,759 inventory plots.


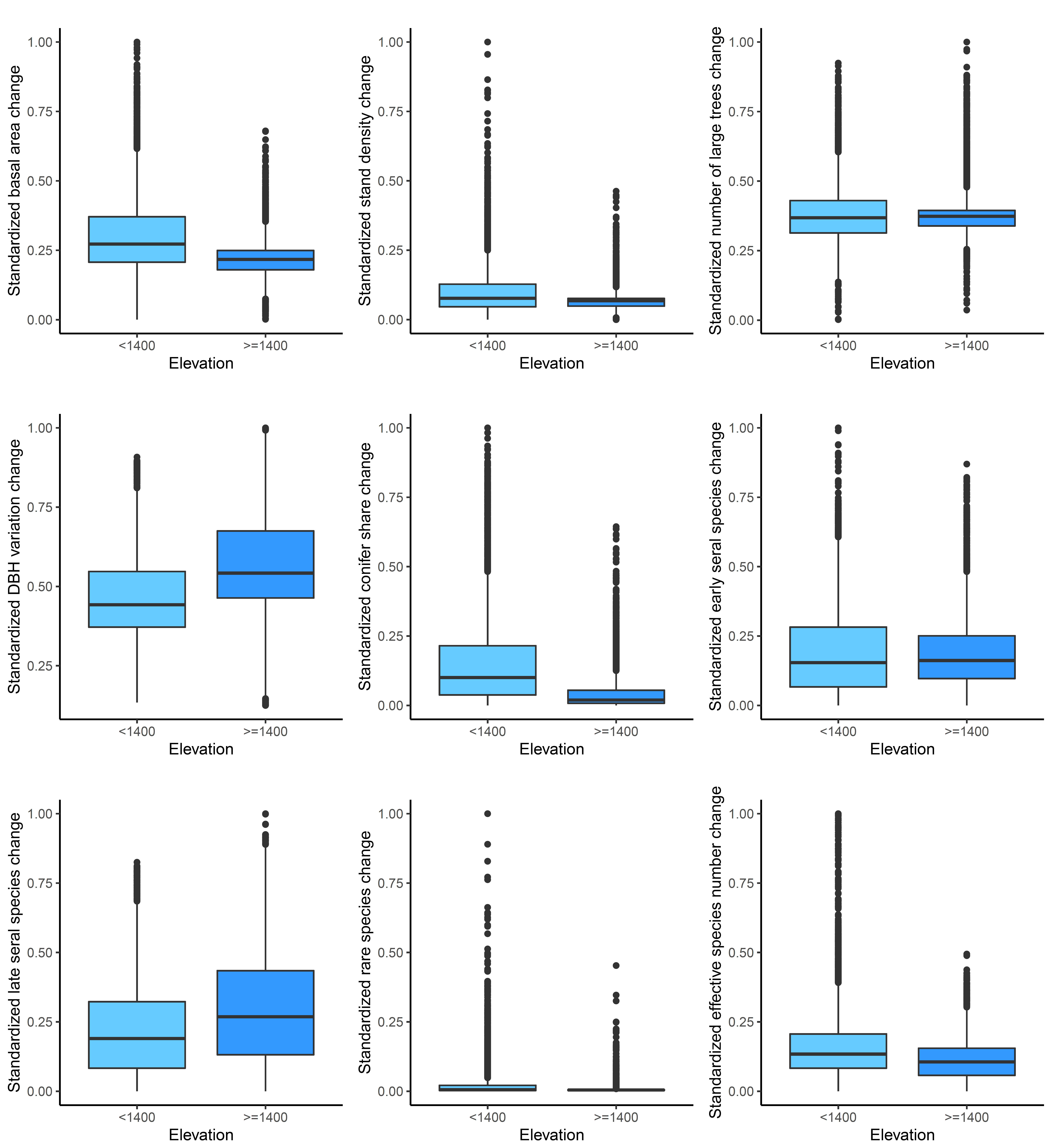
Figure S12: Standardized changes in forest indicators between the year 1984 and 2011 for elevations <1,400 and ≥1,400 m asl. 1,400 m asl. represents the transition between montane and sub-alpine forests.


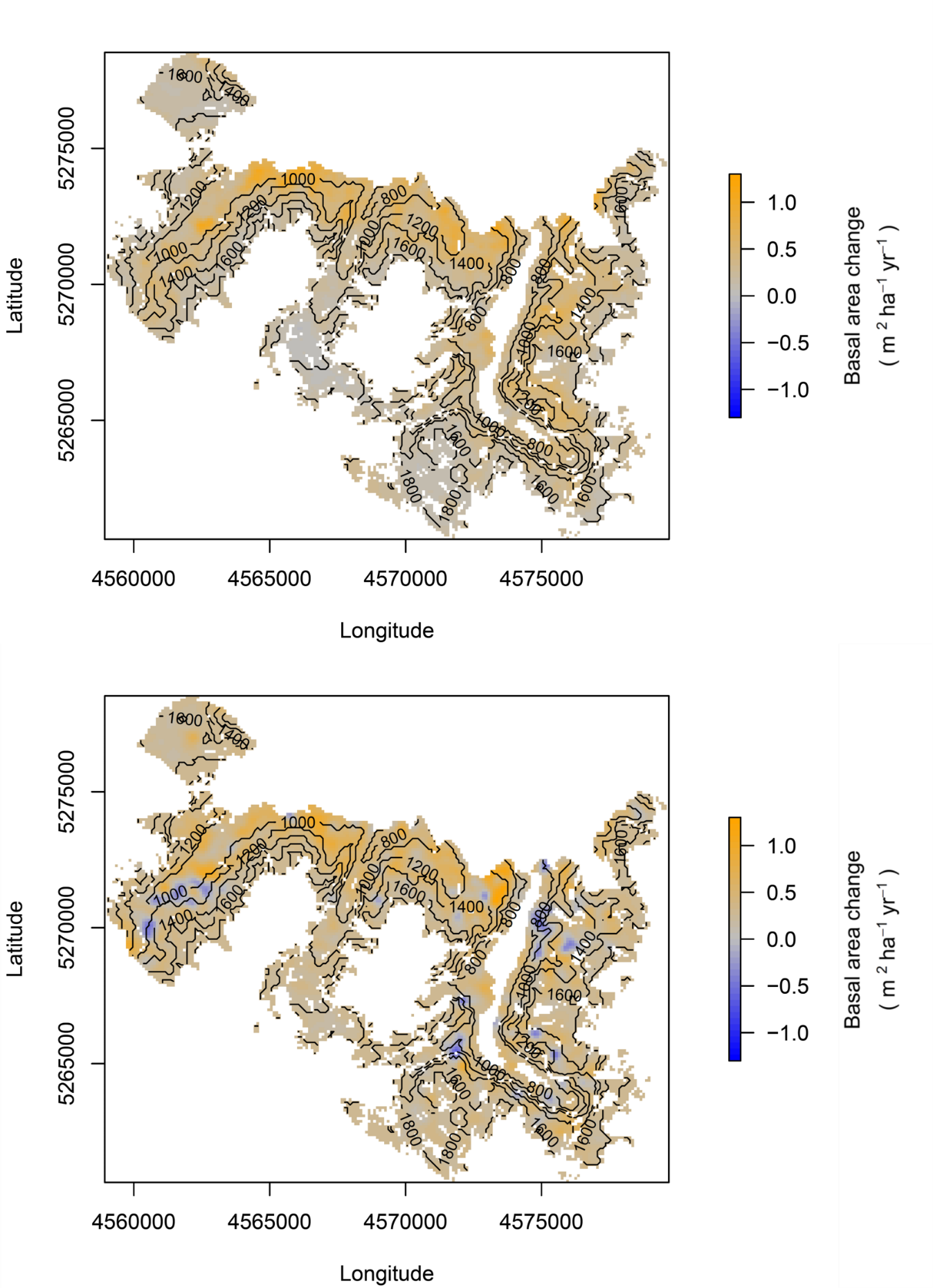


Figure S13: Spatial distribution of absolute annual changes in basal area for the observation periods 1984 – 1996 (upper panel) and 1996 – 2011 (lower panel). Values of 3,759 inventory plots were spatially interpolated on a 100 m grid by means of kriging with spatial autocorrelation determined by semi-variogram models. Isolines indicate elevation asl.


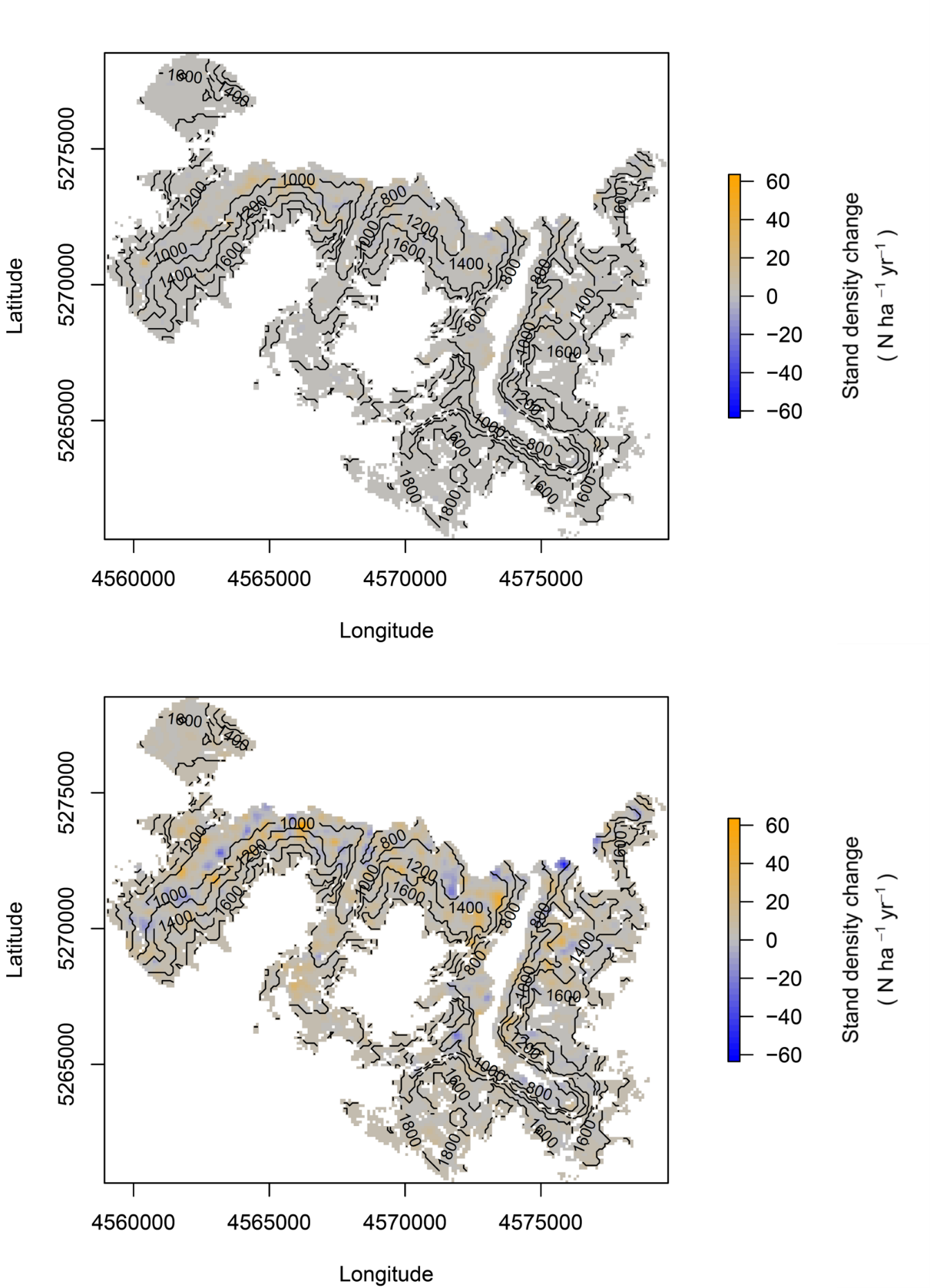


Figure S14: Spatial distribution of absolute annual changes in stand density for the observation periods 1984 – 1996 (upper panel) and 1996 – 2011 (lower panel). Values of 3,759 inventory plots were spatially interpolated on a 100 m grid by means of kriging with spatial autocorrelation determined by semi-variogram models. Isolines indicate elevation asl.


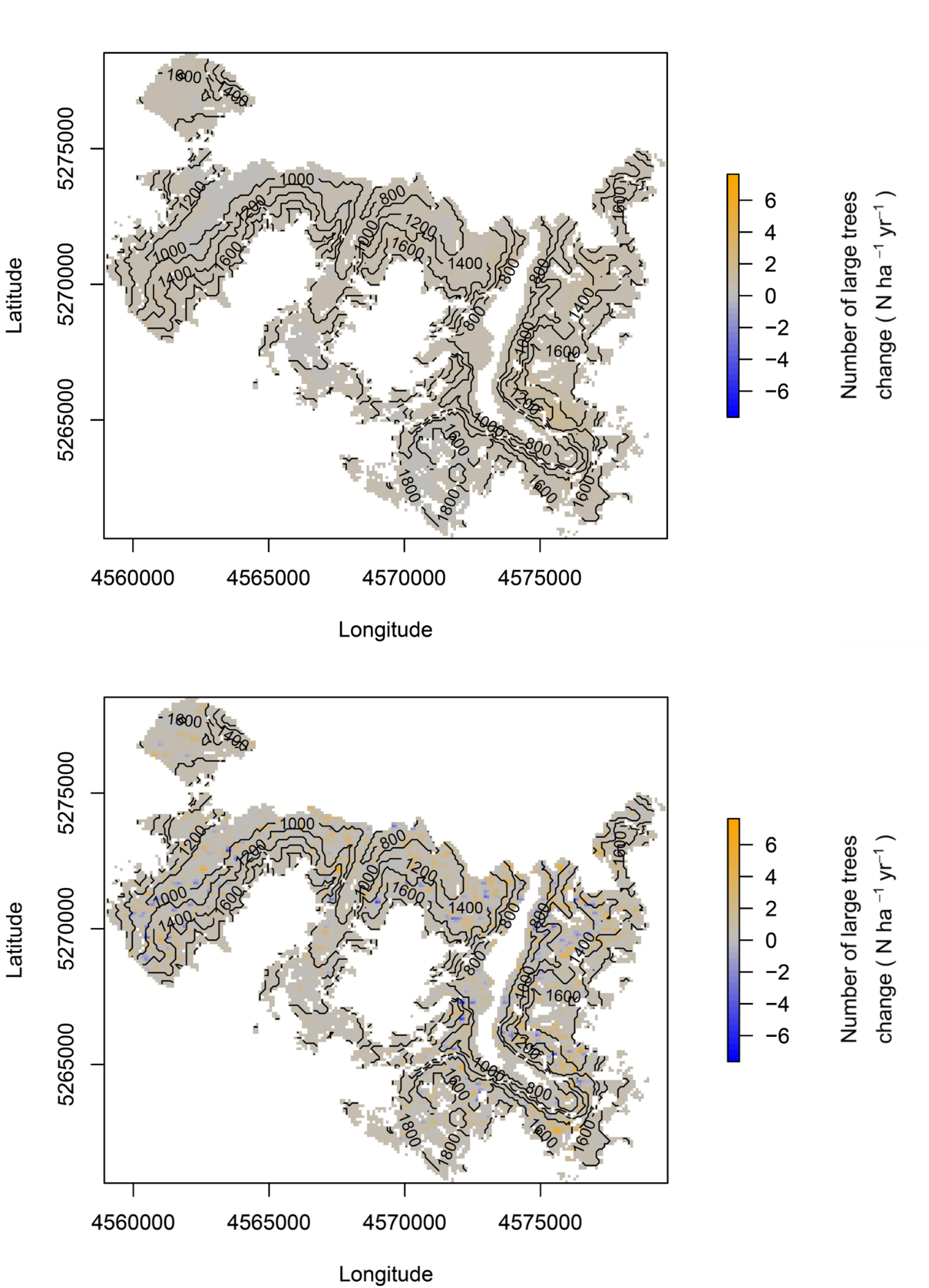


Figure S15: Spatial distribution of absolute annual changes in the number of large trees (DBH > 50 cm) per hectare for the observation periods 1984 – 1996 (upper panel) and 1996 – 2011 (lower panel). Values of 3,759 inventory plots were spatially interpolated on a 100 m grid by means of kriging with spatial autocorrelation determined by semi-variogram models. Isolines indicate elevation asl.


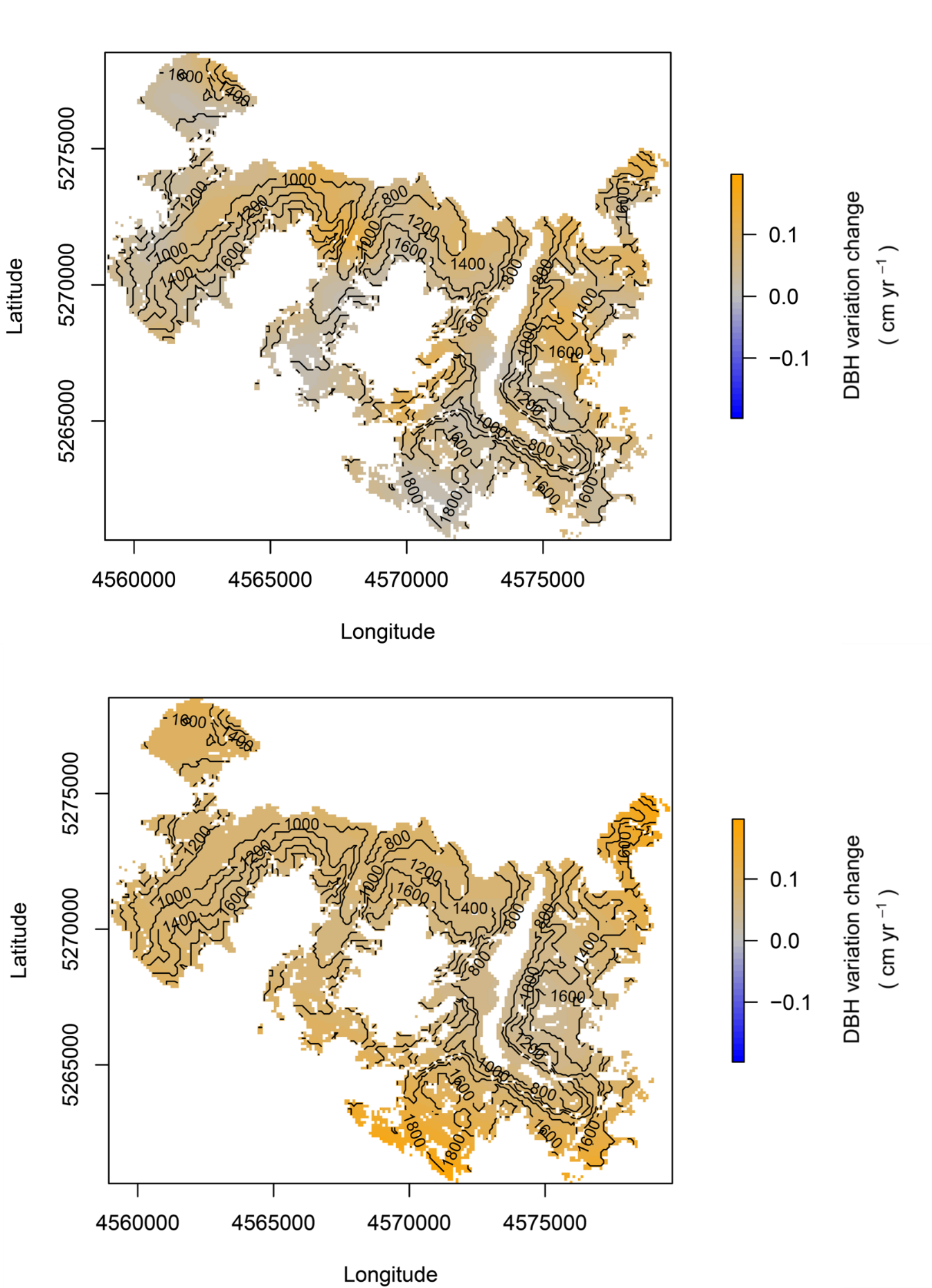


Figure S16: Spatial distribution of absolute annual changes in DBH variation (expressed as standard deviation of DBH) for the observation periods 1984 – 1996 (upper panel) and 1996 – 2011 (lower panel). Values of 3,759 inventory plots were spatially interpolated on a 100 m grid by means of kriging with spatial autocorrelation determined by semi-variogram models. Isolines indicate elevation asl.


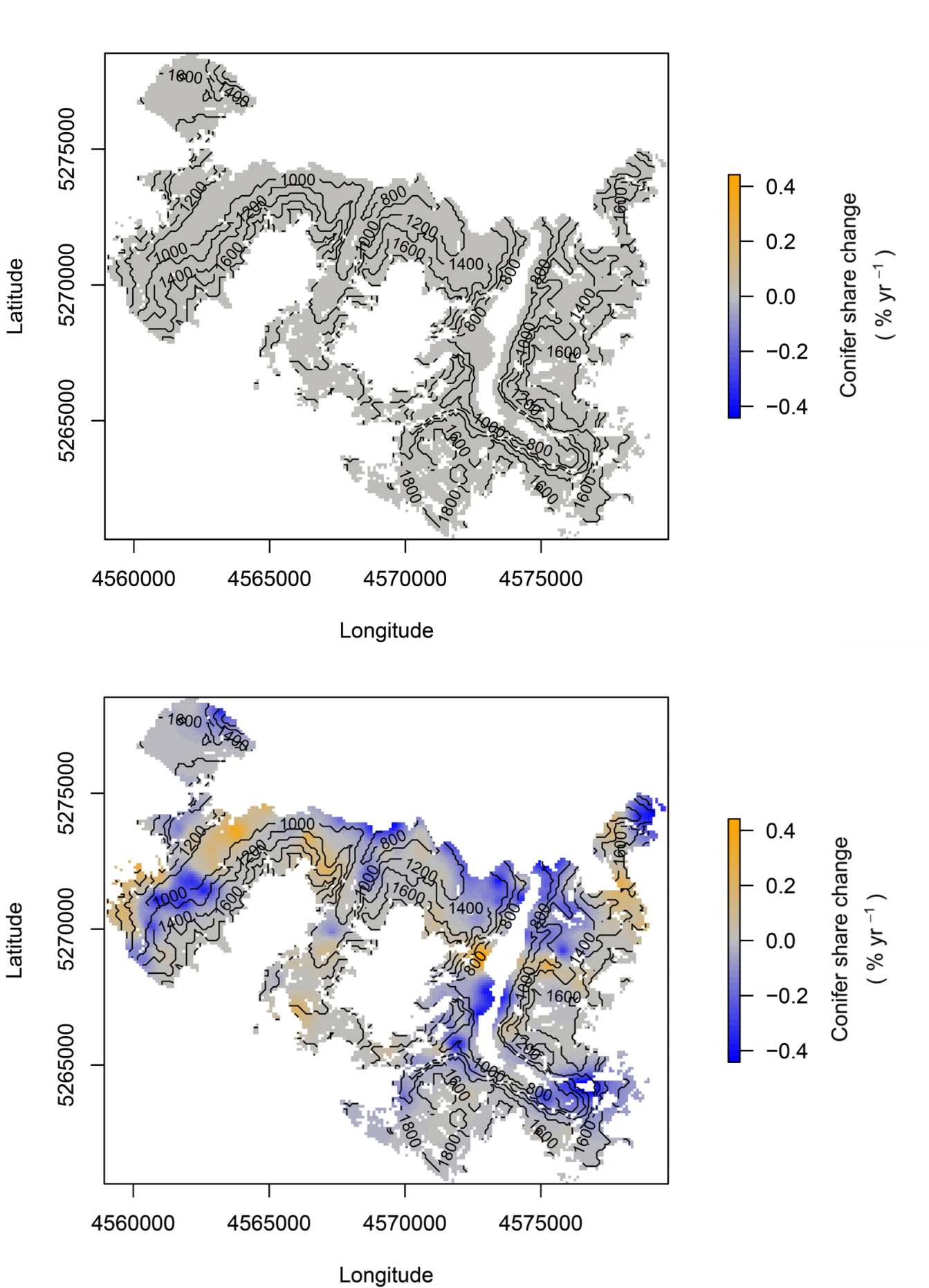


Figure S17: Spatial distribution of absolute annual changes in conifer share for the observation periods 1984 – 1996 (upper panel) and 1996 – 2011 (lower panel). Values of 3,759 inventory plots were spatially interpolated on a 100 m grid by means of kriging with spatial autocorrelation determined by semi-variogram models. Isolines indicate elevation asl.


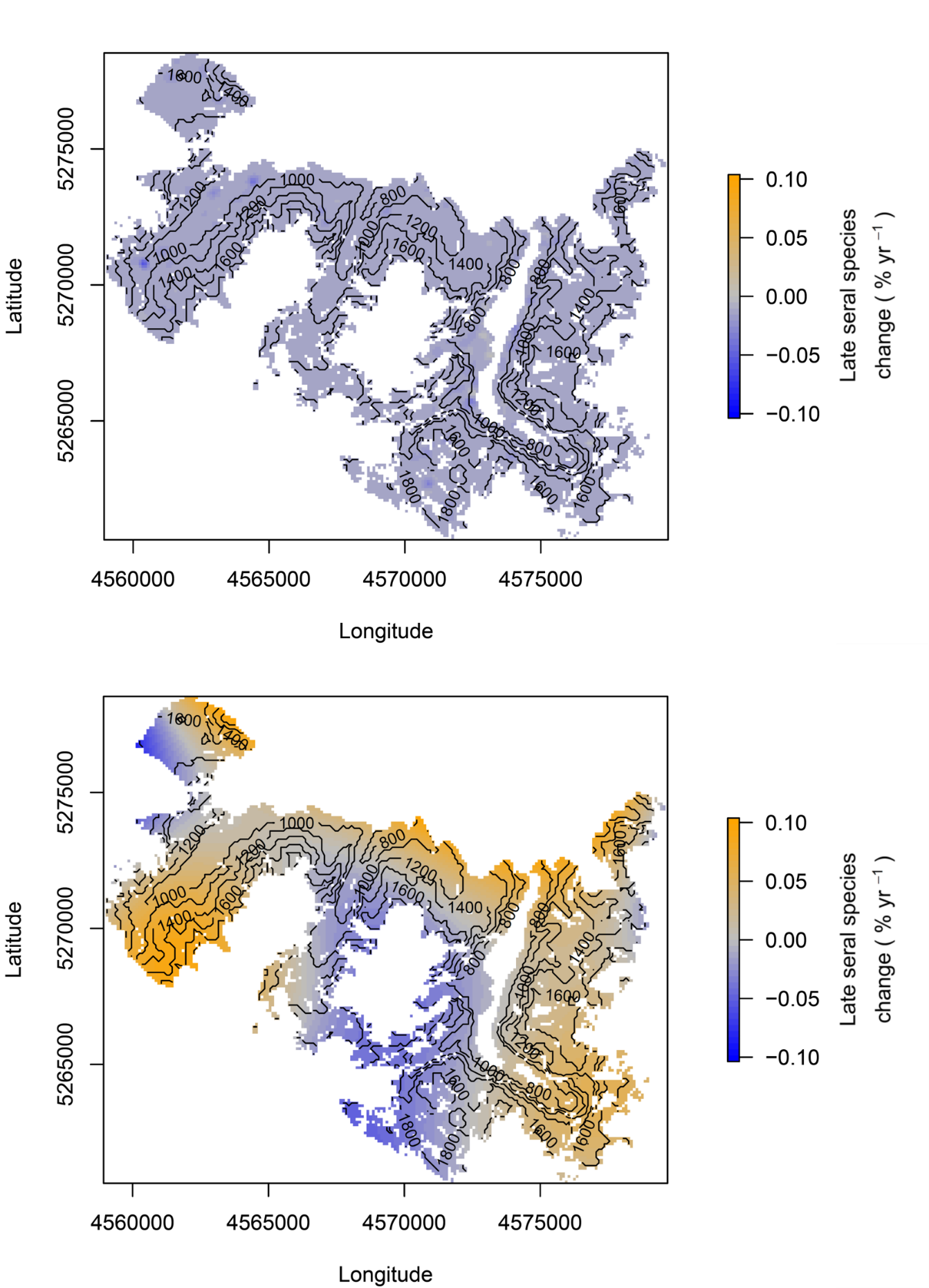


Figure S18: Spatial distribution of absolute annual changes in the share of late seral species for the observation periods 1984 – 1996 (upper panel) and 1996 – 2011 (lower panel). Values of 3,759 inventory plots were spatially interpolated on a 100 m grid by means of kriging with spatial autocorrelation determined by semi-variogram models. Isolines indicate elevation asl.


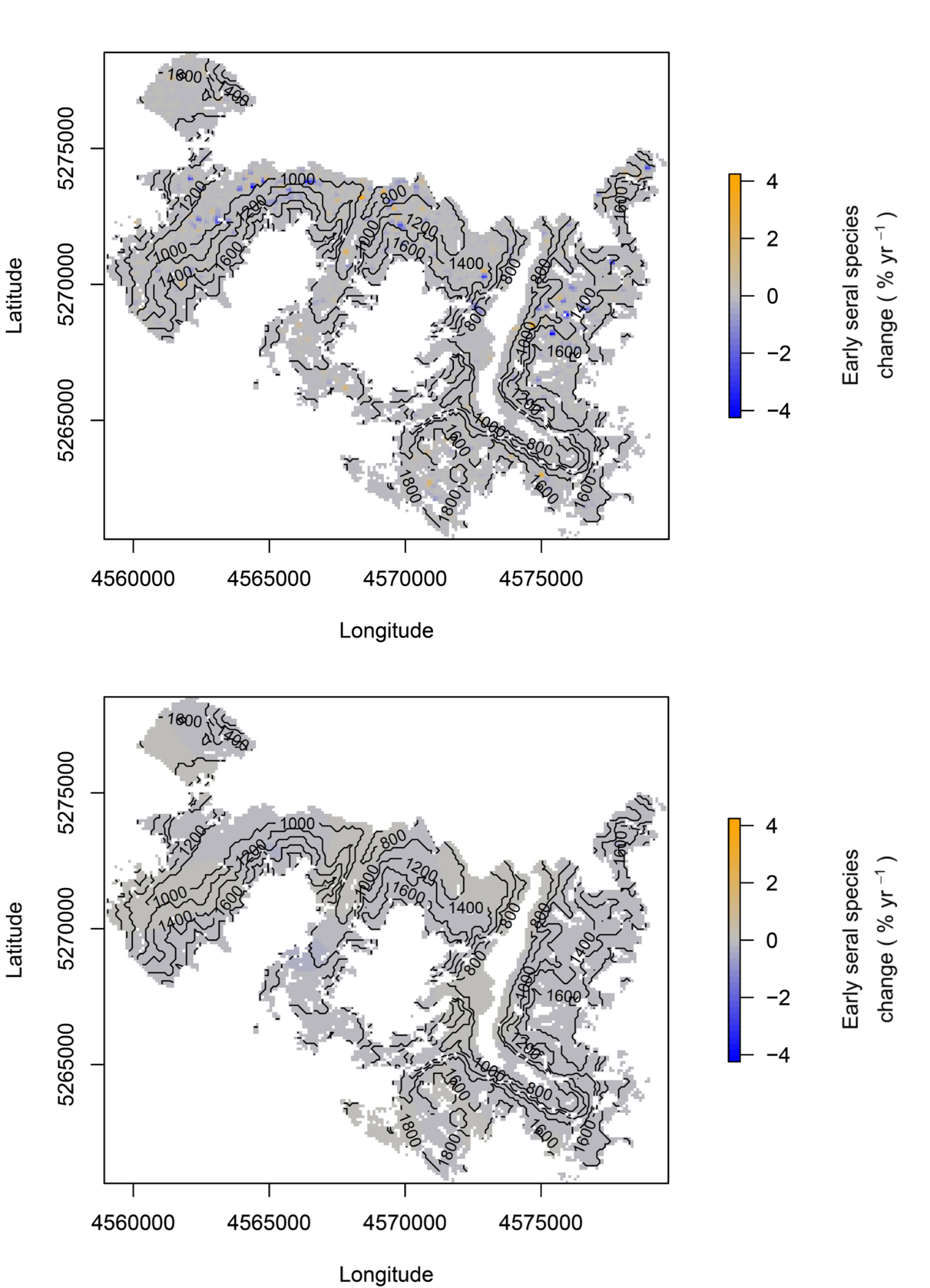


Figure S19: Spatial distribution of absolute annual changes in the share of early seral species for the observation periods 1984 – 1996 (upper panel) and 1996 – 2011 (lower panel). Values of 3,759 inventory plots were spatially interpolated on a 100 m grid by means of kriging with spatial autocorrelation determined by semi-variogram models. Isolines indicate elevation asl.


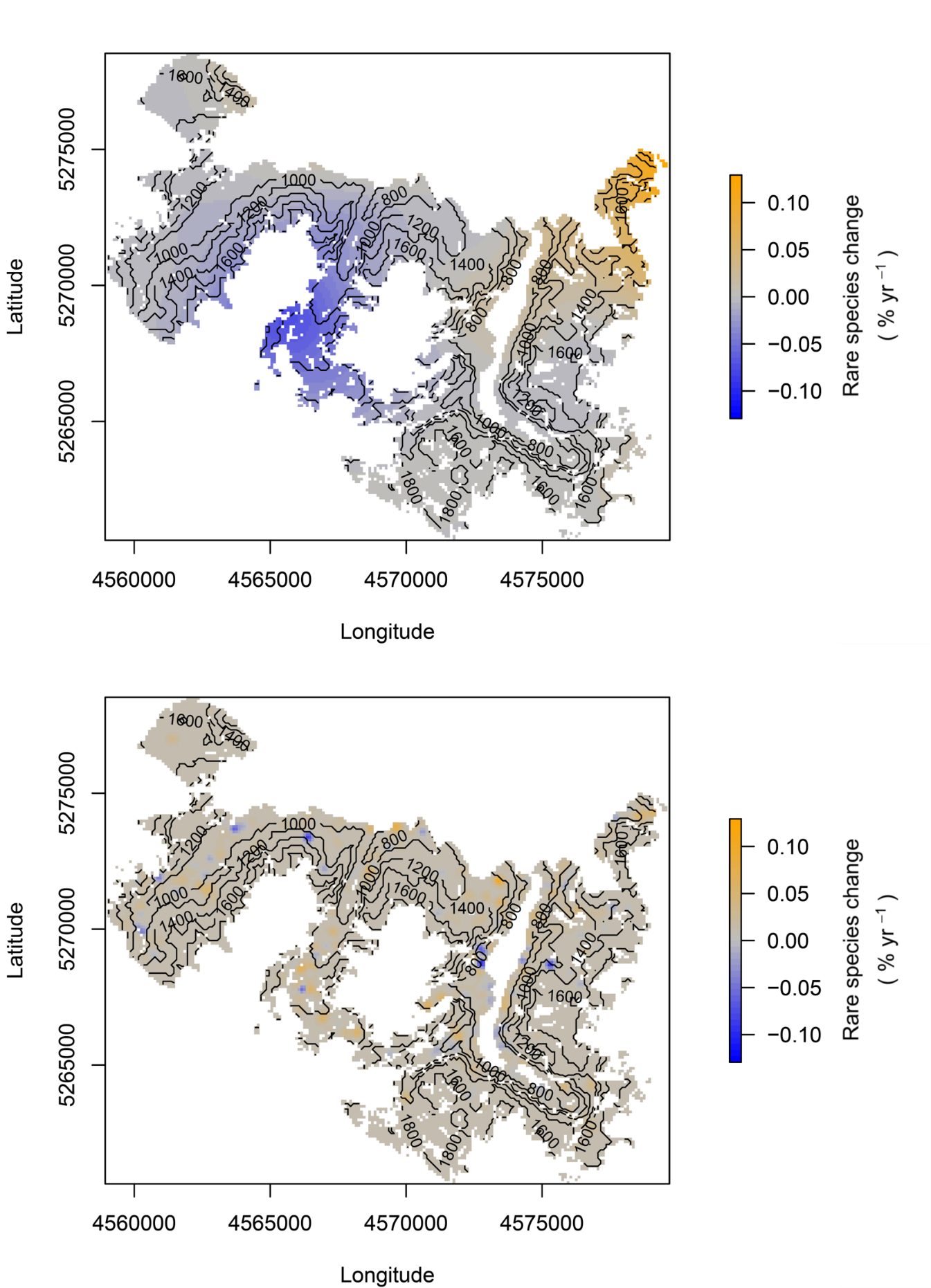


Figure S20: Spatial distribution of absolute annual changes in the share of rare species for the observation periods 1984 – 1996 (upper panel) and 1996 – 2011 (lower panel). Values of 3,759 inventory plots were spatially interpolated on a 100 m grid by means of kriging with spatial autocorrelation determined by semi-variogram models. Isolines indicate elevation asl.


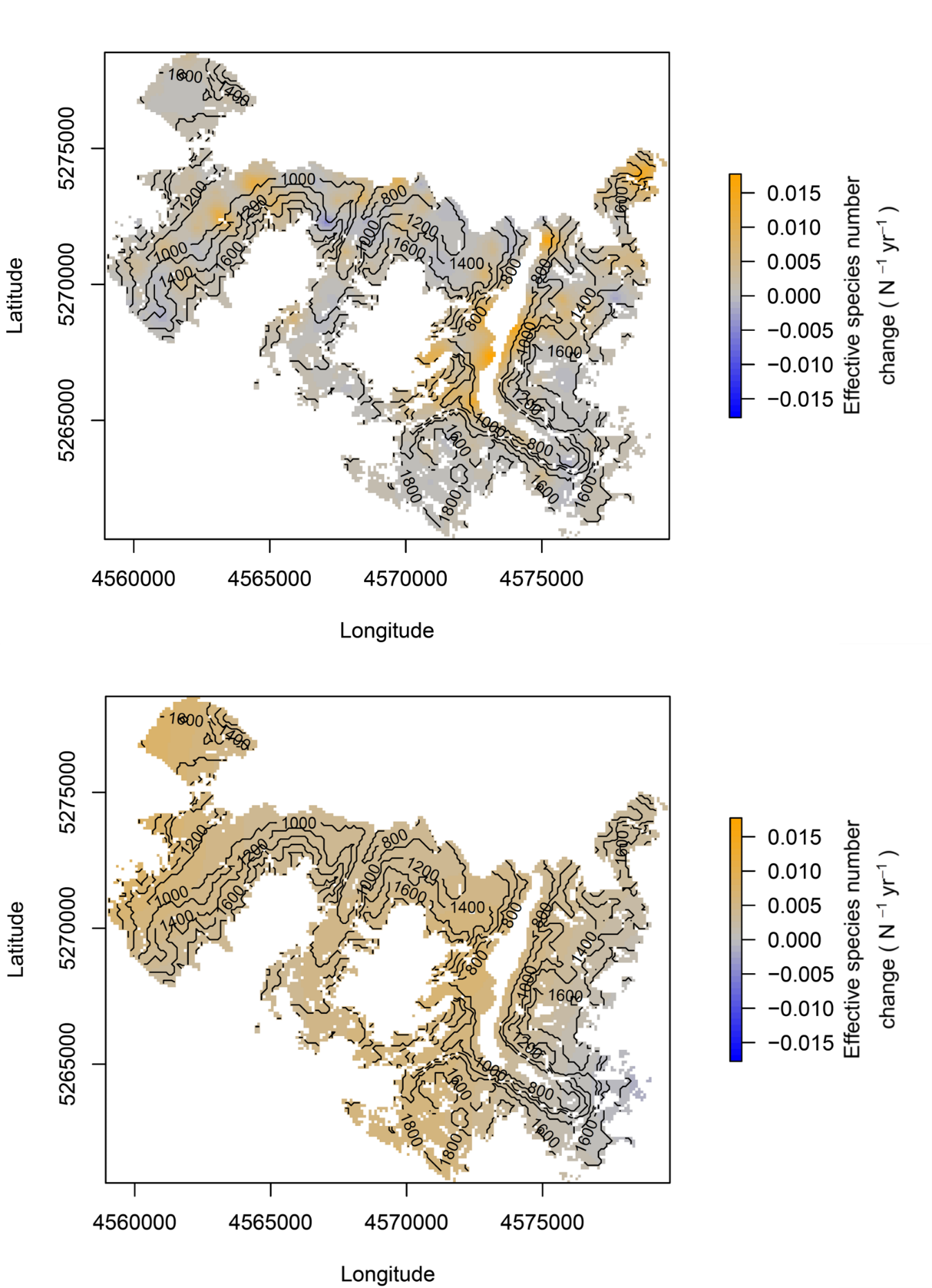


Figure S21: Spatial distribution of absolute annual changes in effective species number (expressed as the exponent of the Shannon-Index) for the observation periods 1984 – 1996 (upper panel) and 1996 – 2011 (lower panel). Values of 3,759 inventory plots were spatially interpolated on a 100 m grid by means of kriging with spatial autocorrelation determined by semi-variogram models. Isolines indicate elevation asl.

**Drivers of forest change**


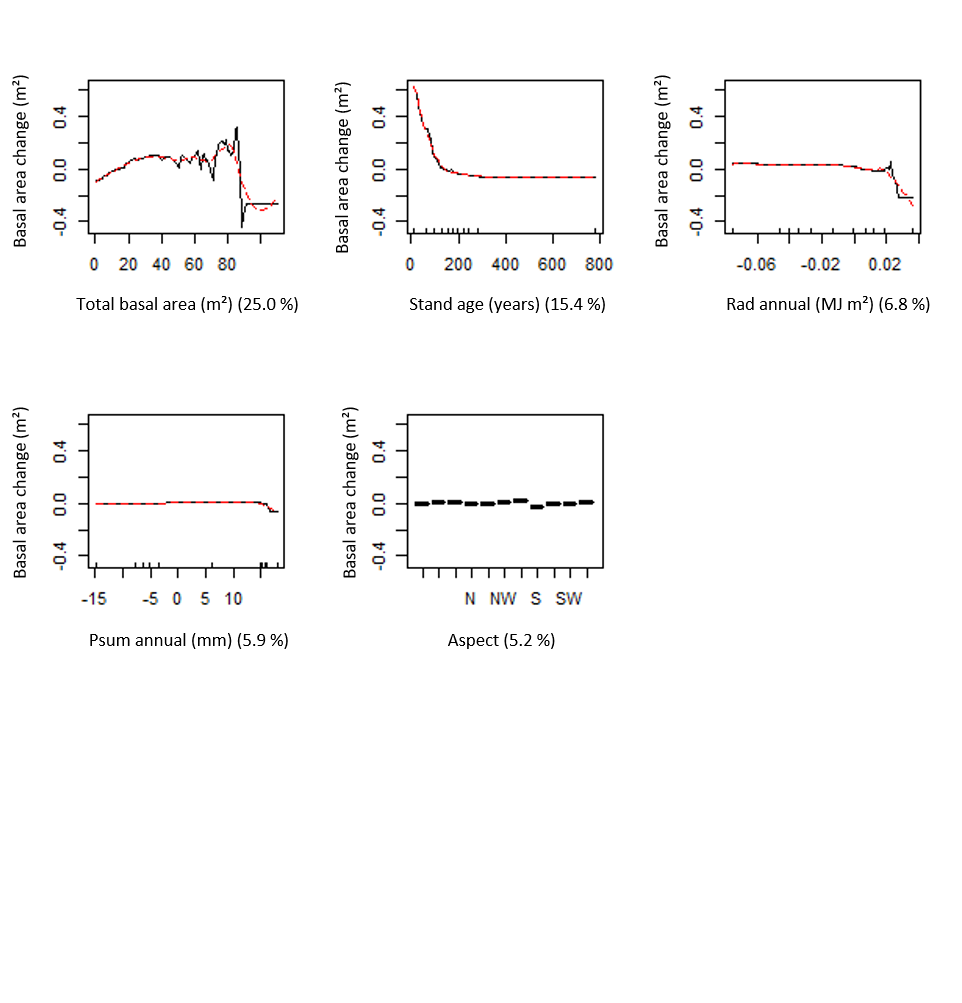


Figure S22: Partial dependence plots of boosted regression tree (BRT) models presenting the fitted functions of indicators explaining changes in basal area. Shown are covariates with a relative influence (in parentheses) of ≥ 5% on the final model. Smoothed curves are visualized with dashed red lines over the partial dependence functions to facilitate interpretation.


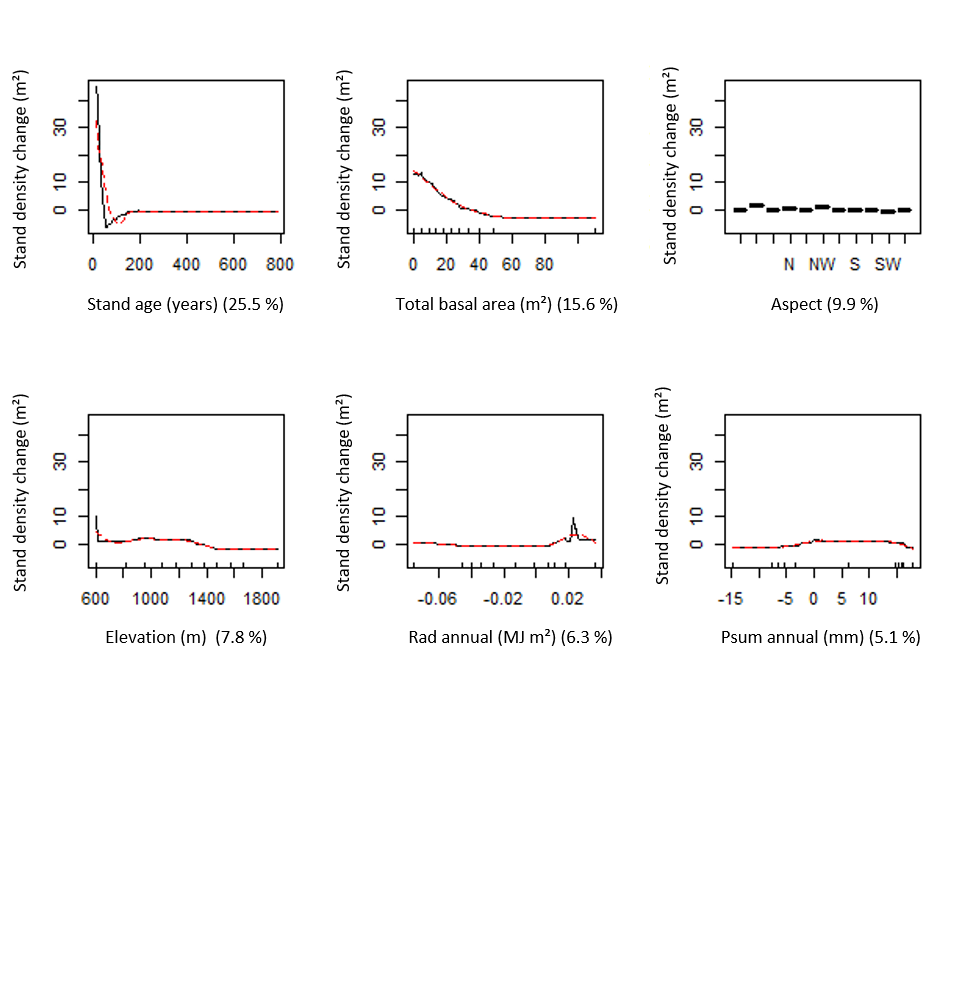
Figure S23: Partial dependence plots of boosted regression tree (BRT) models presenting the fitted functions of indicators explaining changes in stand density. Shown are covariates with a relative influence (in parentheses) of ≥ 5% on the final model. Smoothed curves are visualized with dashed red lines over the partial dependence functions to facilitate interpretation.


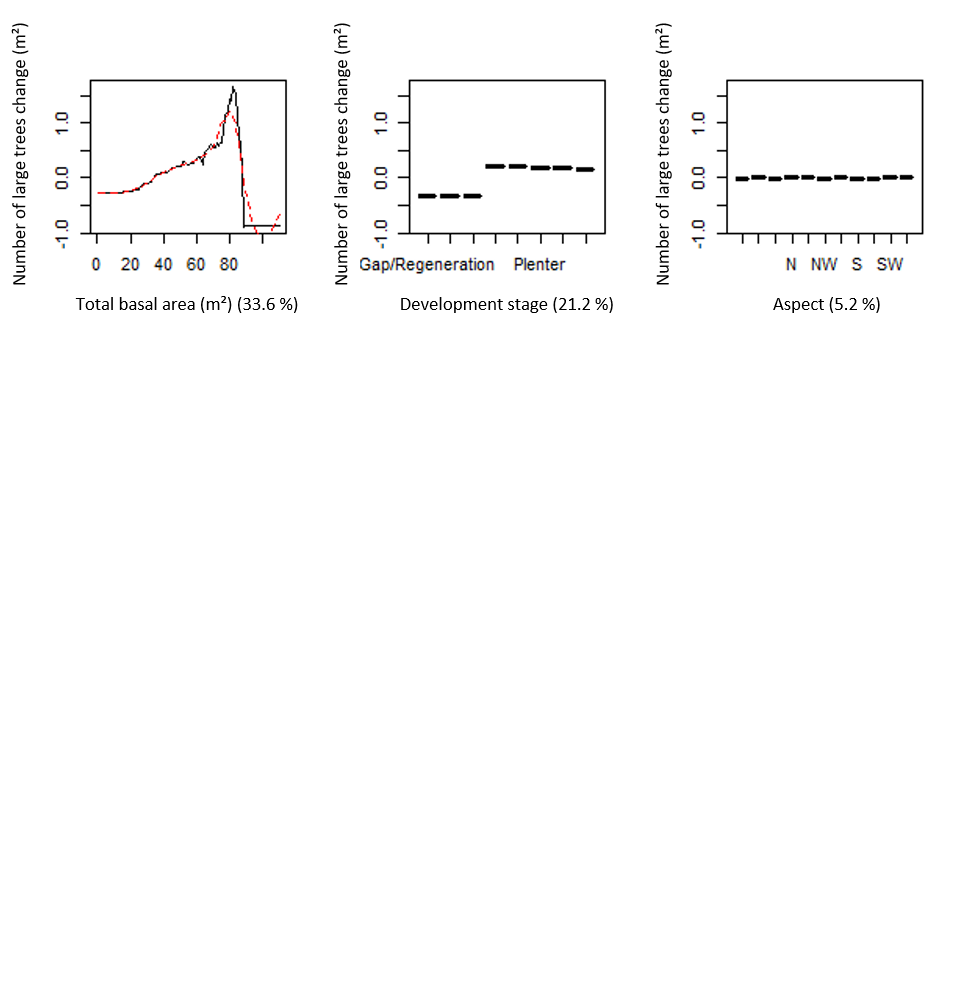


Figure S24: Partial dependence plots of boosted regression tree (BRT) models presenting the fitted functions of indicators explaining changes in large tree (dbh > 50 cm) density. Shown are covariates with a relative influence (in parentheses) of ≥ 5% on the final model. Smoothed curves are visualized with dashed red lines over the partial dependence functions to facilitate interpretation.


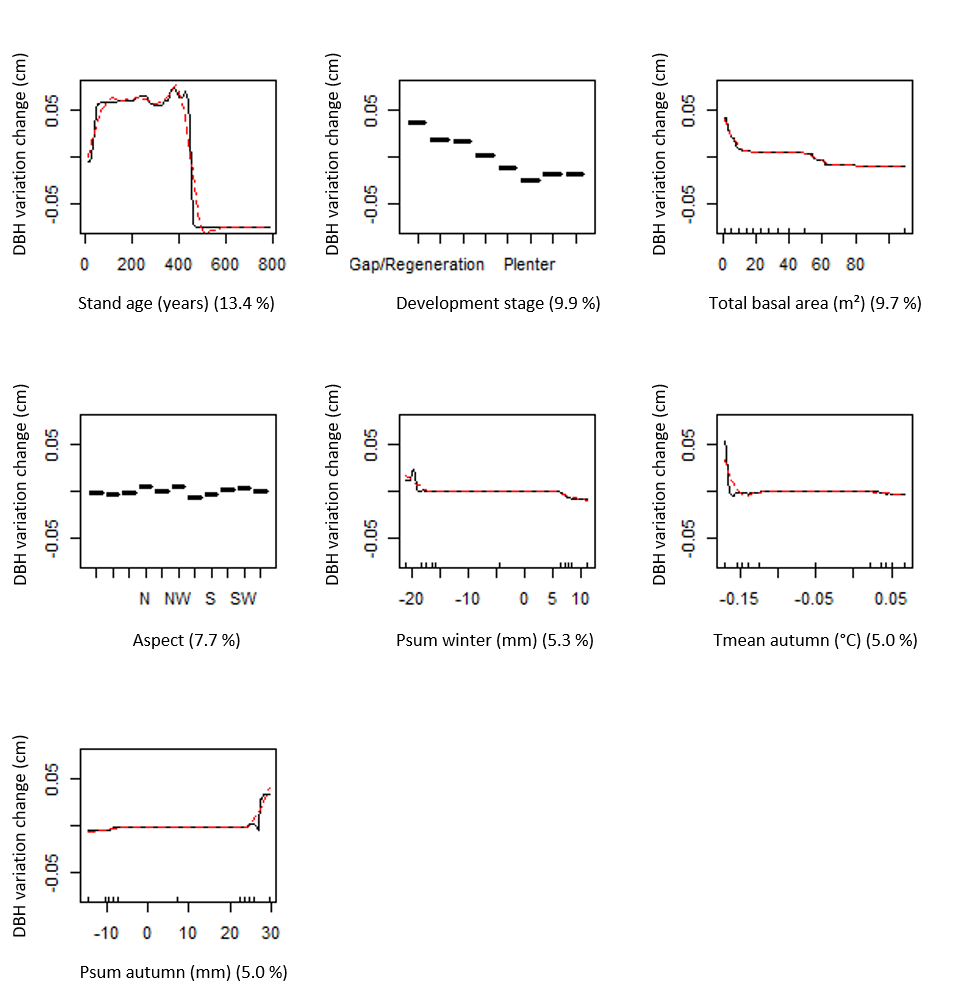


Figure S25: Partial dependence plots of boosted regression tree (BRT) models presenting the fitted functions of indicators explaining changes in the variation (SD) of tree dbh. Shown are covariates with a relative influence (in parentheses) of ≥ 5% on the final model. Smoothed curves are visualized with dashed red lines over the partial dependence functions to facilitate interpretation.


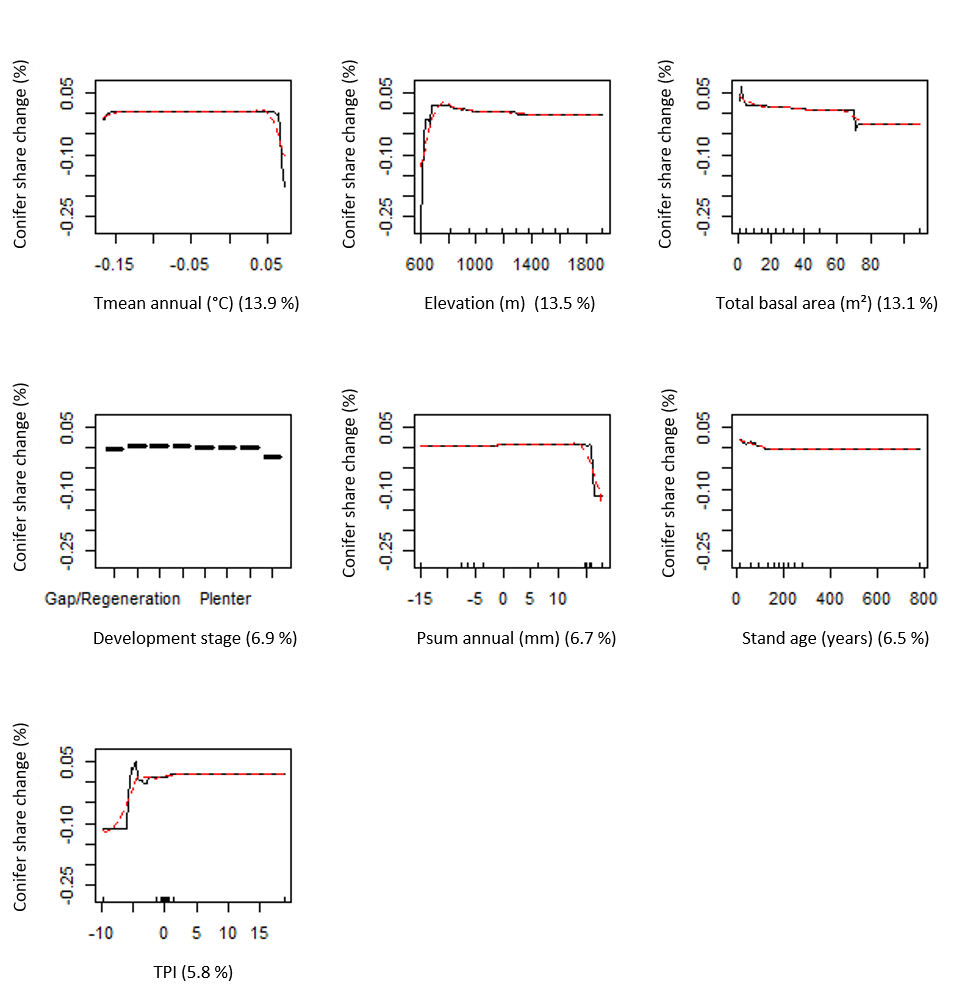


Figure S26: Partial dependence plots of boosted regression tree (BRT) models presenting the fitted functions of indicators explaining changes in the proportion of conifers. Shown are covariates with a relative influence (in parentheses) of ≥ 5% on the final model. Smoothed curves are visualized with dashed red lines over the partial dependence functions to facilitate interpretation.


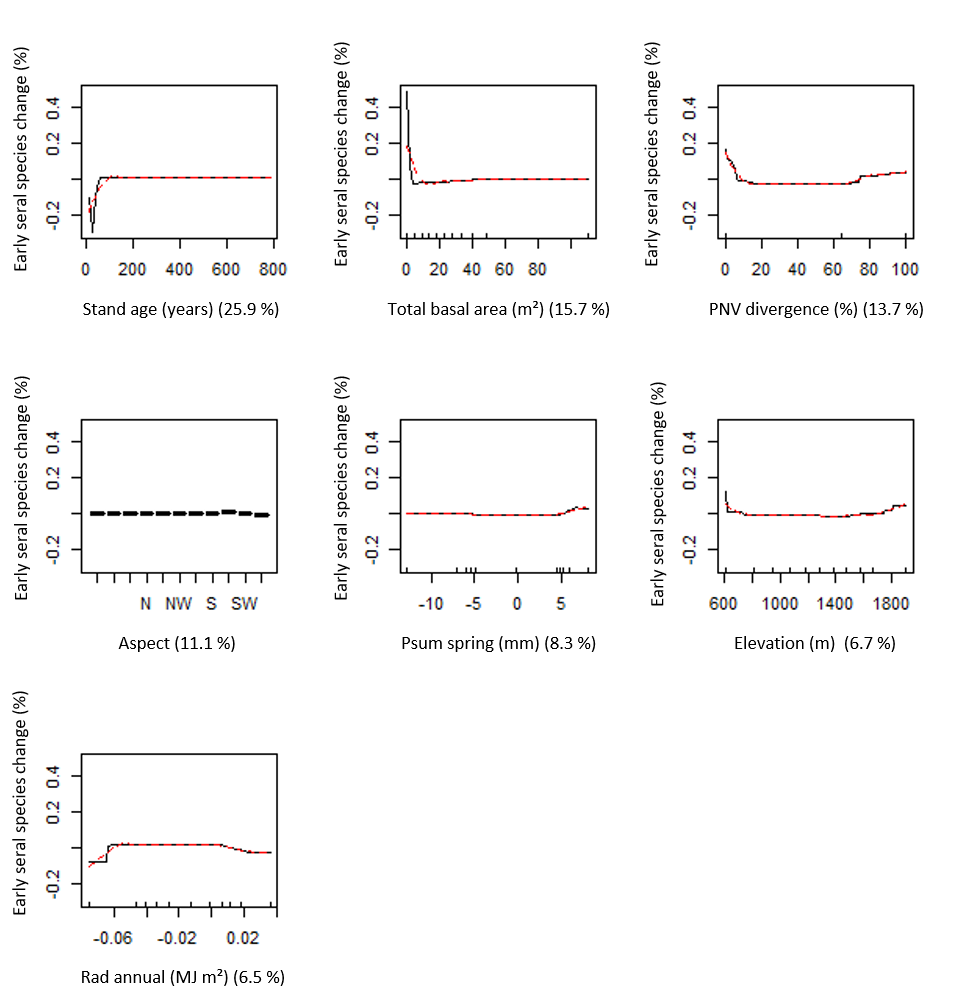


Figure S27: Partial dependence plots of boosted regression tree (BRT) models presenting the fitted functions of indicators explaining changes in the proportion of early seral species. Shown are covariates with a relative influence (in parentheses) of ≥ 5% on the final model. Smoothed curves are visualized with dashed red lines over the partial dependence functions to facilitate interpretation.


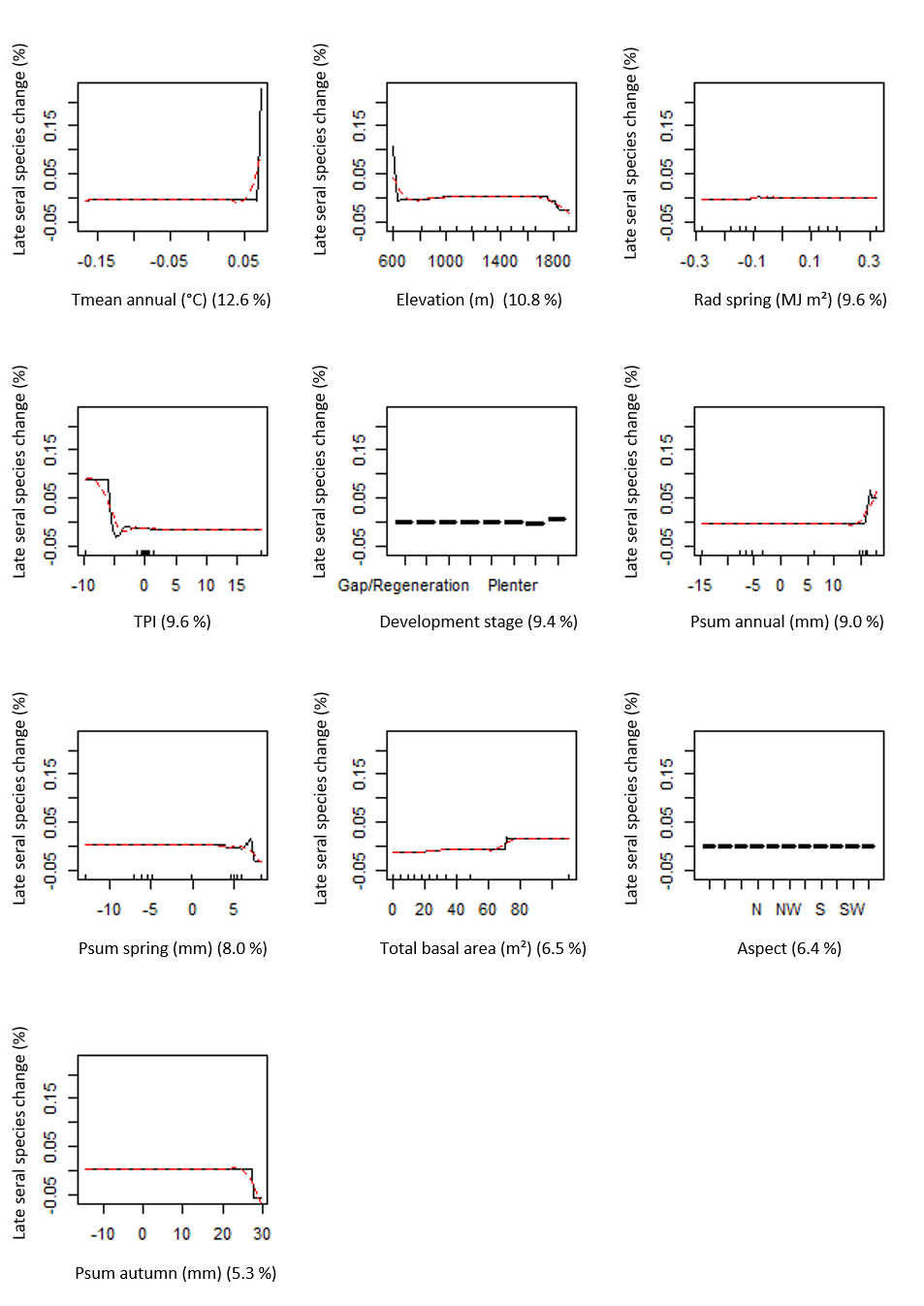


Figure S28: Partial dependence plots of boosted regression tree (BRT) models presenting the fitted functions of indicators explaining changes in the proportion of late seral species. Shown are covariates with a relative influence (in parentheses) of ≥ 5% on the final model. Smoothed curves are visualized with dashed red lines over the partial dependence functions to facilitate interpretation.


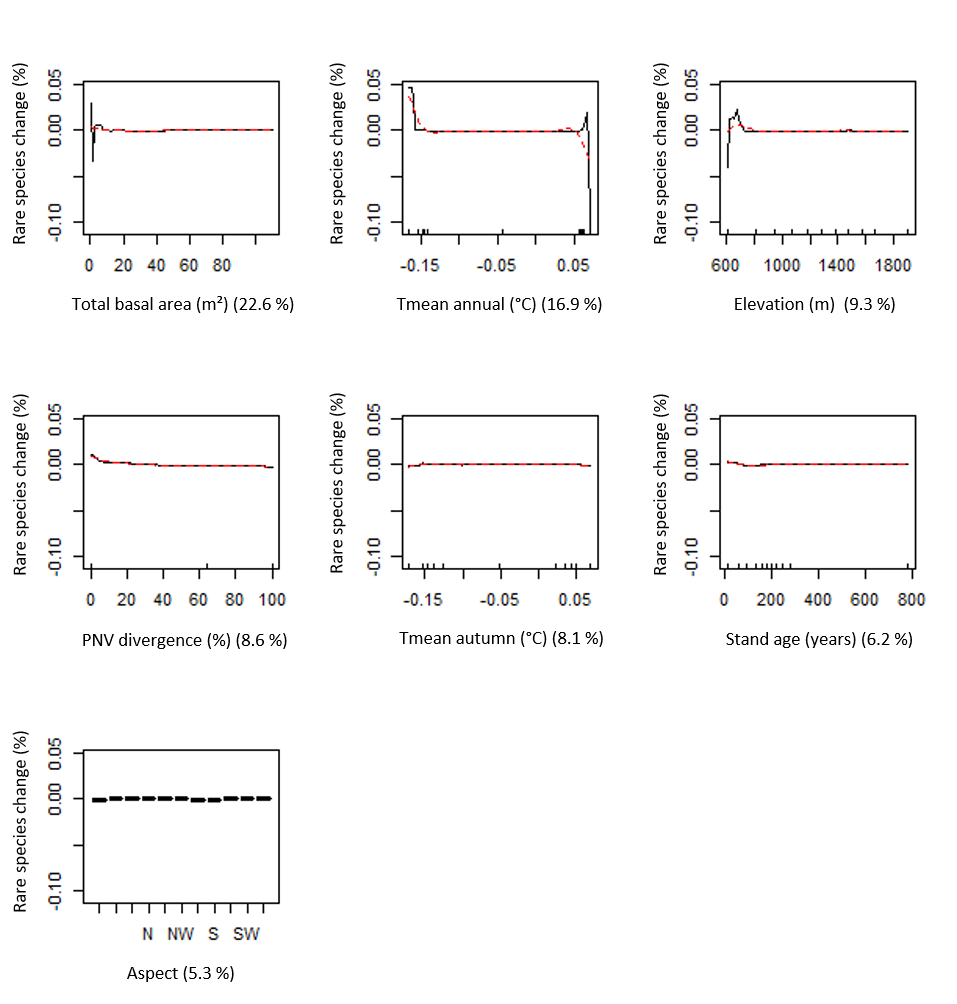
Figure S29: Partial dependence plots of boosted regression tree (BRT) models presenting the fitted functions of indicators explaining changes in the proportion of rare species. Shown are covariates with a relative influence (in parentheses) of ≥ 5% on the final model. Smoothed curves are visualized with dashed red lines over the partial dependence functions to facilitate interpretation.


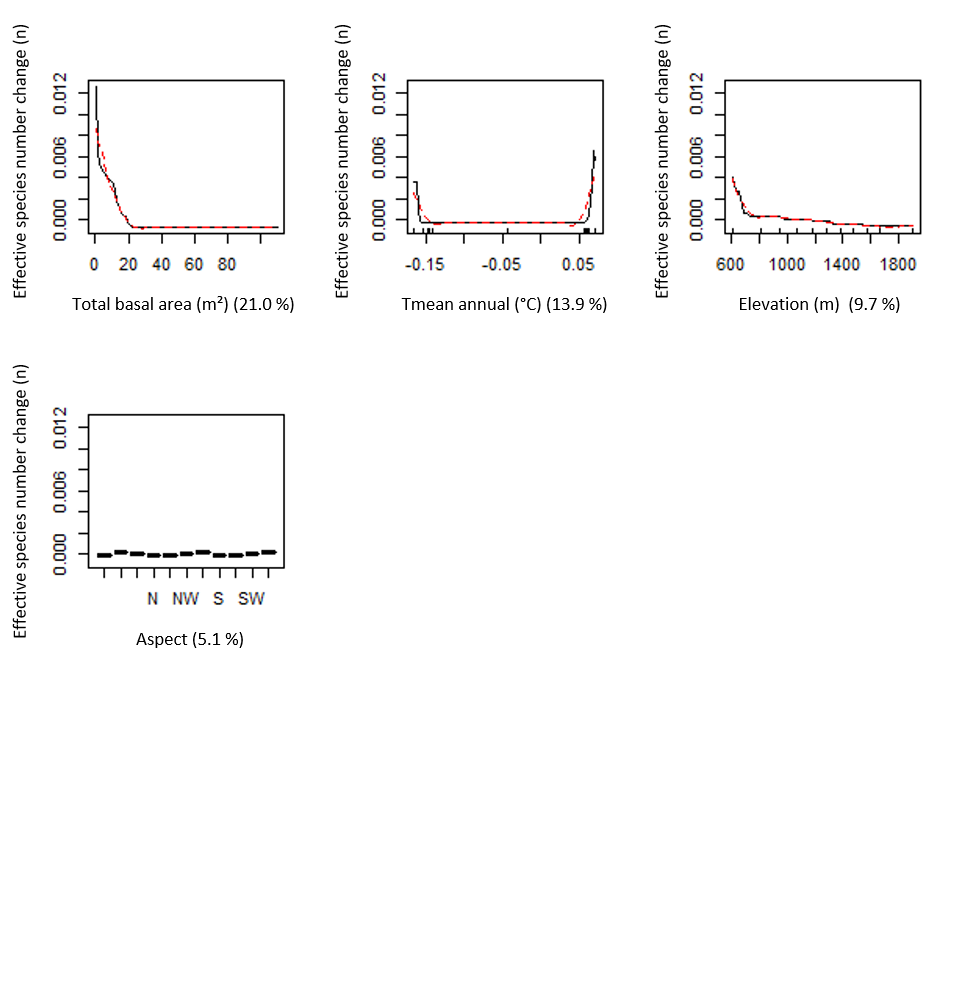


Figure S30: Partial dependence plots of boosted regression tree (BRT) models presenting the fitted functions of indicators explaining changes in the effective tree species number. Shown are covariates with a relative influence (in parentheses) of ≥ 5% on the final model. Smoothed curves are visualized with dashed red lines over the partial dependence functions to facilitate interpretation.


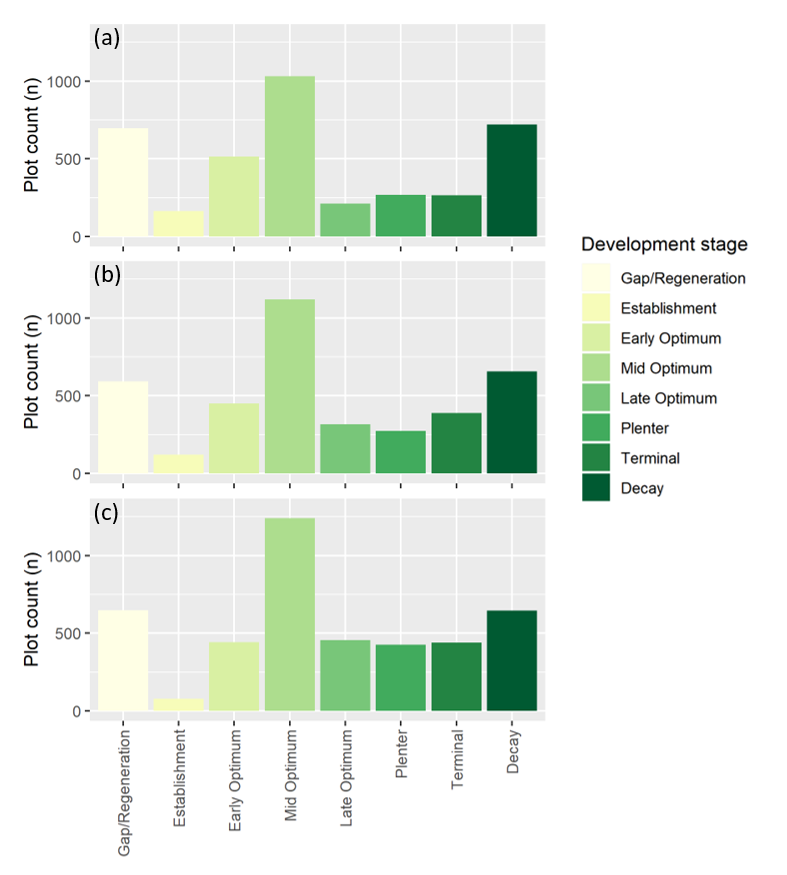


Figure S31: Forest development stages at Berchtesgaden National Park. Presented are development stages at the time of the first (1983-1985), second (1995-1997), and third (2010-2012) forest inventory. The total number of inventory plots is 3,759. Development stages were classified based on Zenner and others (2016).


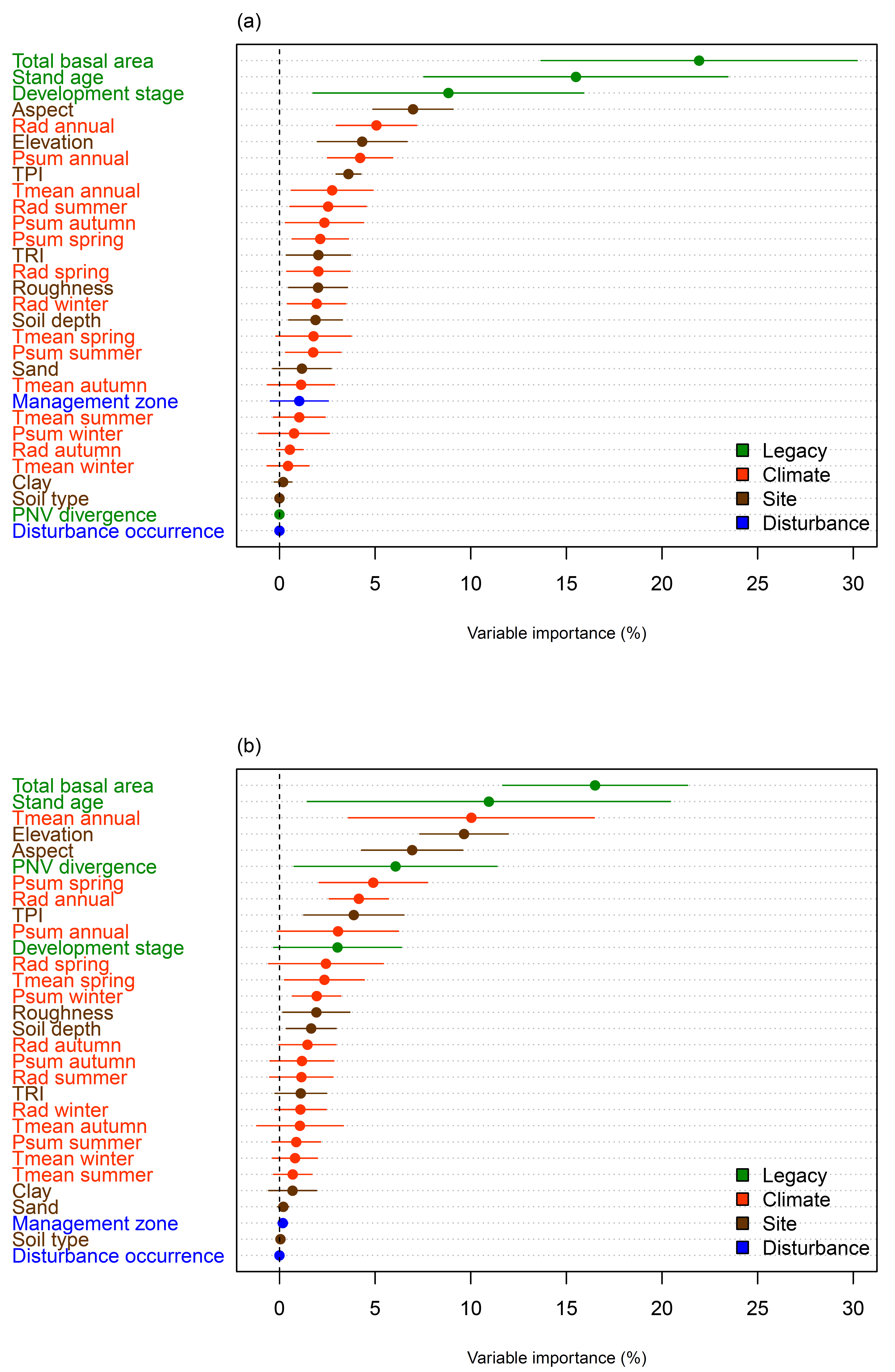


Figure S32: Relative importance of drivers explaining forest change. Shown are the (a) drivers of structural change, and (b) drivers of compositional change. Presented are weighted averages (dots) and confidence intervals (whiskers) for the relative importance of drivers explaining the combined changes of four structural and five compositional attributes across two inventory periods (1984-1996, 1996-2011) based on BRT models. The relative importance of each driver was weighted by the variance explained in cross-validation.
